# Hemodynamic and metabolomic responses to infusion of GLP-1 agonist exenatide in pulmonary arterial hypertension

**DOI:** 10.1172/jci.insight.202660

**Published:** 2026-05-22

**Authors:** Chinthaka B. Samaranayake, Marili Niglas, Nicoleta Baxan, Alexander Kempny, Ali Ashek, Michael Gatzoulis, Laura C. Price, Konstantinos Dimopoulos, Martin R. Wilkins, Stephen Wort, Christopher J. Rhodes, Lan Zhao, Colm McCabe

**Affiliations:** 1 National Pulmonary Hypertension Service, Royal Brompton Hospital, Guys and St. Thomas’ NHS Trust, London, United Kingdom.; 2 National Heart and Lung Institute, Imperial College, London, United Kingdom.

**Keywords:** Cardiology, Pulmonology, Cardiovascular disease, Metabolomics

## Abstract

Preclinical studies suggest beneficial effects of GLP-1 agonists in pulmonary arterial hypertension (PAH). This first-in-disease study evaluated acute hemodynamic effects of GLP-1 agonist, exenatide administered i.v. in patients with idiopathic PAH and CTEPH as well as in a PAH rodent model. Seventeen patients (9 idiopathic PAH) received an exenatide infusion during right heart catheterization, which included multisite sampling for circulating metabolites. Acute effects of exenatide were also assessed by cardiac magnetic resonance imaging in monocrotaline (MCT) PAH and control rats. In the clinical study, exenatide was well tolerated, reduced mean pulmonary artery pressure (45 ± 15 mmHg versus 40 ± 18 mmHg), and improved cardiac index (2.1 ± 0.6 L/min versus 2.4 ± 0.9 L/min/m^2^) and pulmonary vascular resistance (7.8 ± 8.0 WU versus 5.9 ± 5.0 WU) across all patients. Right ventricular (RV) contractility and afterload improved in a subset of patients undergoing pressure-volume measurements. In an exploratory metabolomics analysis, 47 metabolite levels changed after exenatide infusion, predominantly in free fatty acid pathways. Six metabolites with prognostic relevance in PAH within myocardial glycolytic and lipid oxidation pathways were also altered after exenatide. In MCT rats, exenatide improved RV stroke-volume, RV ejection fraction, and RV-arterial coupling. These findings support the further evaluation of exenatide within chronic studies as a potentially novel pulmonary vasodilator therapy.

## Abstract 2

GLP-1 agonist, exenatide causes acute relaxation of lung blood vessles in pulmonary arterial hypertension

## Introduction

Pulmonary arterial hypertension (PAH) is characterized by progressive arteriolar vasoconstriction and vascular remodeling in the pulmonary circulation leading to impaired performance of the right ventricle (RV). Established drug treatments in PAH target reversal of pulmonary vasoconstriction (1); however, RV dysfunction, which governs clinical prognosis in PAH, frequently persists with a 5-year survival approximating 65% (2–4). Novel agents such as sotatercept, which target the TGF-β pathway, have shown highly promising results even when administered in combination with existing therapies, although long-term outcome data are currently lacking (5, 6). This underscores the need for novel therapies for PAH, beyond the current treatment pool.

Glucagon-like peptide-1 (GLP-1), a naturally occurring incretin peptide released from intestinal L-cells, has wide-ranging effects on glucose metabolism mediated by the GLP-1 receptor (GLP-1R). GLP-1R has a broad tissue distribution that includes the heart and lung vasculature and is used therapeutically at scale to target insulin resistance and obesity (7). Of potential relevance, increased GLP-1R expression has been demonstrated in smooth muscle cells of pulmonary arteries and arterioles (8, 9), and both native GLP-1 and synthetic GLP-1 agonists including exenatide demonstrate a vasorelaxant effect across several vascular beds, potentially mediated by nitric oxide (10–14). Moreover, there is a recognized association between insulin resistance (IR) and PAH, with studies demonstrating that patients with IR have worse World Health Organization (WHO) functional class and right ventricular function compared with insulin-sensitive patients with PAH (15).

We investigated the acute hemodynamic effects of the GLP-1 agonist, exenatide administered intravenously in idiopathic PAH (IPAH) and chronic thromboembolic pulmonary hypertension (CTEPH), 2 first-in-disease patient cohorts. Multisite plasma sampling was undertaken in an exploratory analysis to assess metabolomic responses to exenatide infusion across the heart and lung vasculature. In a further in vivo PAH animal model, changes in cardiac volumes and contractile RV function in response to exenatide were evaluated by CMR.

## Results

### Patient demographics

Seventeen patients (*n* = 9 IPAH and *n* = 8 CTEPH) were prospectively recruited and included in the hemodynamic study. The median age was 55 years (interquartile range 32), and all were in WHO function class II (*n* = 10) or III (*n* = 7). Patients had to be clinically stable with no evidence of decompensated right heart failure. Baseline demographics, clinical characteristics, and pulmonary hemodynamics of patients are summarized in Table 1.

### Safety and tolerability

I.v. infusion of exenatide was well tolerated with the exception of 2 patients (11.8%) who developed nausea that necessitated interruption after 20 and 25 minutes of the infusion, respectively. A trend toward a reduction in serum blood glucose level was observed with exenatide (Supplemental Figure 1; supplemental material available online with this article; https://doi.org/10.1172/jci.insight.202660DS1).

### Pulmonary hemodynamics

**Figure 1:**
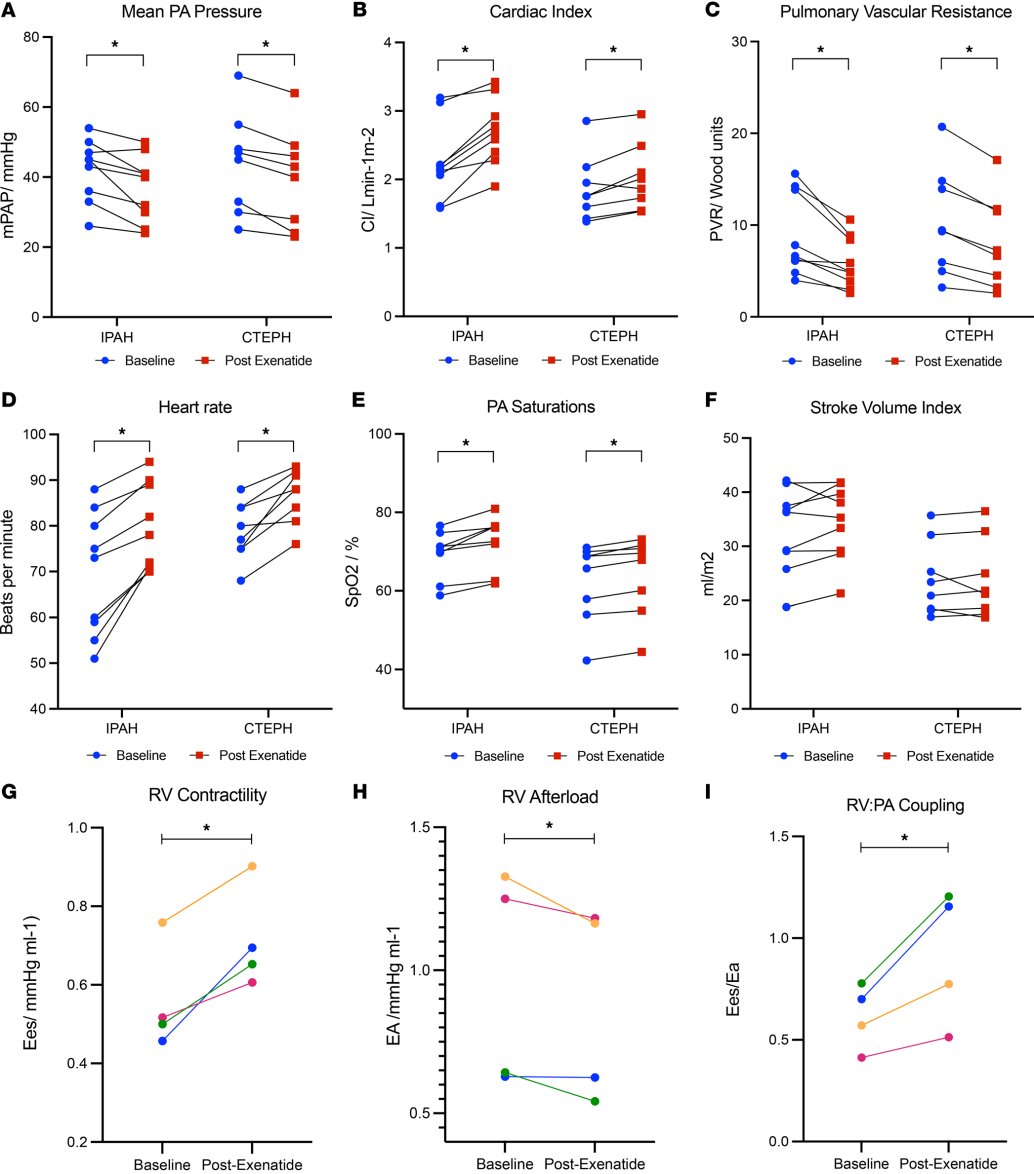
Change in pulmonary hemodynamics before and after exenatide in patients with IPAH (*n* = 9) and CTEPH (*n* = 8). (**A**–**C**) Shown are the change in mean pulmonary artery pressure (**A**), cardiac index (**B**), pulmonary vascular resistance (**C**), heart rate (**D**), pulmonary artery saturation (**E**), stroke volume index (**F**), right ventricular contractility (*n* = 4) (**G**), right ventricular afterload (*n* = 4) (**H**), and ventricular vascular coupling (**I**). *P < 0.05. The *P* value was determined using the paired sample Wilcoxon signed-rank test (**A**–**I**).

Changes in systemic and pulmonary hemodynamics before and after exenatide infusion are shown in Figure 1 and are further summarized in Supplemental Tables 1 and 2. Significant changes were seen in mean pulmonary artery pressure (–11.1%, *P* = 0.03 in IPAH and –10.8%, *P* = 0.01 in CTEPH), cardiac output (CO; +29.5%, *P* < 0.01 in IPAH and +22.9%, *P* < 0.01 in CTEPH), and pulmonary vascular resistance (–27.9%, *P* = 0.02 in IPAH and –25.5%, *P* < 0.01 in CTEPH) in response to exenatide (Figure 1, A–C). Increase in heart rate was observed in both patients with IPAH and those with CTEPH (Figure 1D). PA saturations improved by 7.0% (*P* < 0.01) in IPAH and 4.5% (*P* = 0.04) in CTEPH (Figure 1E). Stroke volume index (SVi) increased in 7 of 9 patients with IPAH (Figure 1F); however, across all patients with IPAH and CTEPH, the effect of exenatide on stroke volume (SV) was neutral (*P* = 0.17) (Supplemental Tables 1 and 2). Mean systemic arterial pressure was unaffected by exenatide (data not shown).

### Pressure-volume measurements

Four patients with IPAH underwent RV pressure-volume catheterization measurements. RV functional characteristics before and after exenatide infusion are summarized in Supplemental Table 3. Improvement in both RV end-systolic elastance (*P* = 0.022) and effective pulmonary arterial elastance (*P* = 0.043) resulted in improved right ventricular-arterial coupling with exenatide (Figure 1, G–I).

### Plasma metabolomics

#### Effect of exenatide on metabolites (nontargeted analysis).

**Figure 2:**
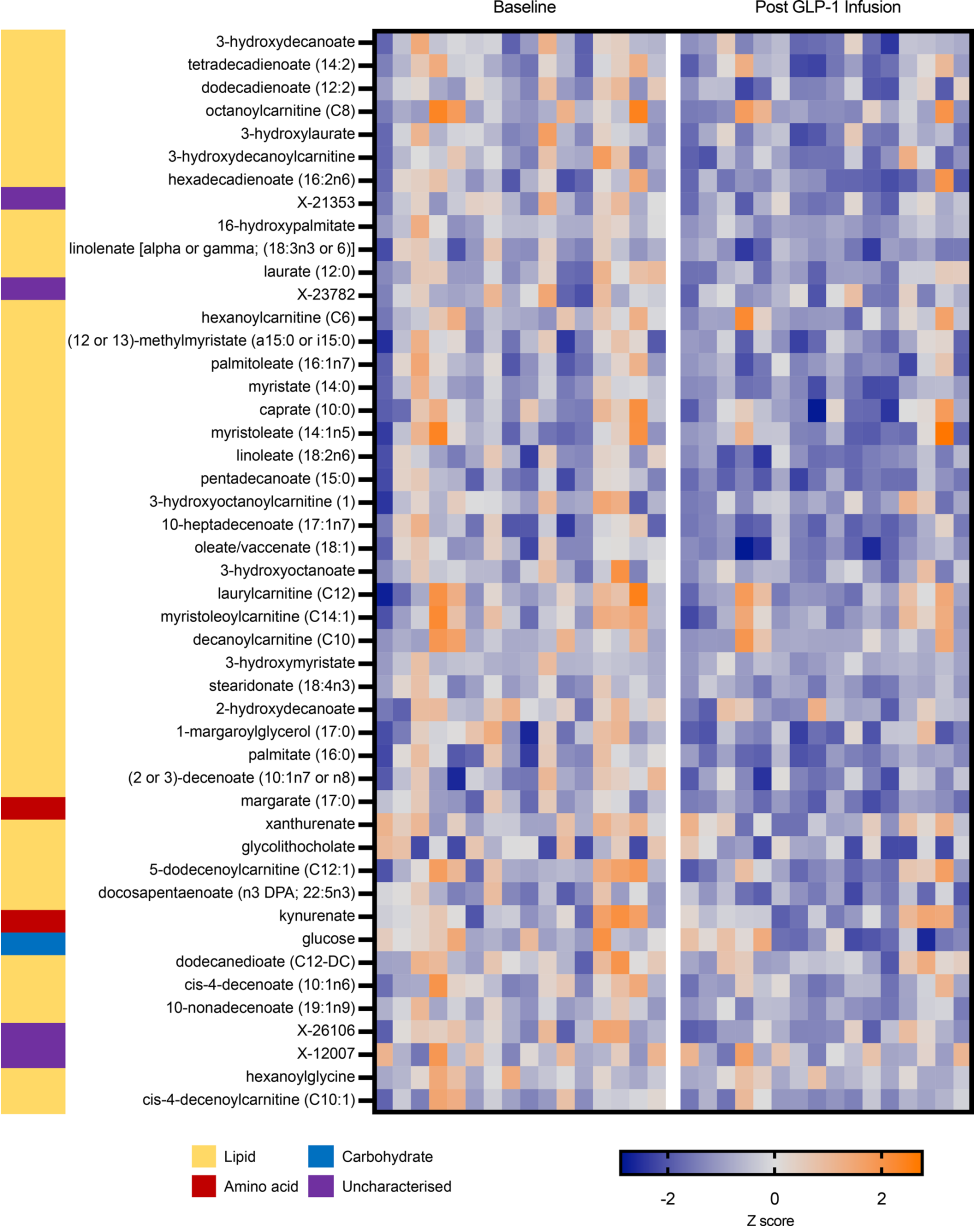
Heatmap demonstrating the change in levels of 47 metabolites before and after exenatide sampled at the radial artery. All the metabolites had a significant change (FDR *q* < 0.05) least significant change. Metabolite levels were normalized by Box-Cox transformations and processed as previously described (16).

A significant change in plasma metabolite levels before and after exenatide infusion was seen in 165 metabolites (Supplemental Figure 2), with 47 reaching FDR-corrected significance (FDR *q* < 0.05) (Figure 2). Among the 47 metabolites reaching FDR-significance (FDR *q* < 0.001) following exenatide, significant enrichment was seen in metabolites of the Lipid super pathway. Out of 135 metabolic subpathways, 6 were enriched (FDR *q* < 0.001) for metabolites that changed significantly following exenatide infusion including Long Chain Monounsaturated Fatty Acids, Long Chain Polyunsaturated Fatty Acids, Acyl Carnitines, Long Chain Saturated Fatty Acids, Monohydroxy Fatty Acids, and Medium Chain Fatty Acids. Detailed values for the mean change in metabolite levels before and after exenatide infusion are shown in Supplemental Table 4.

#### Effect of exenatide on metabolites with prognostic importance in PAH.

**Figure 3:**
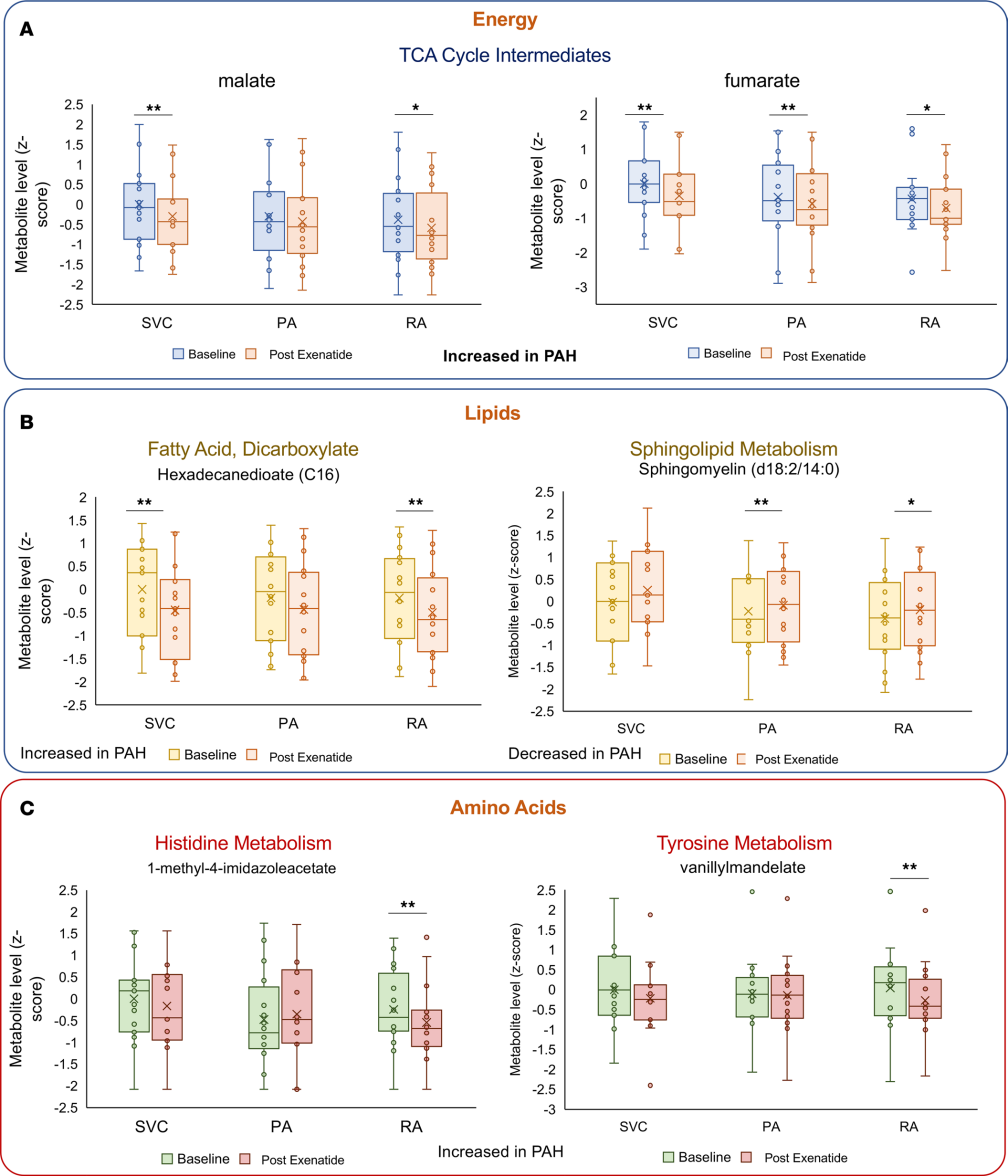
Change in metabolites with known prognostic importance in IPAH. (**A**–**C**) Exenatide administration associated with significant changes in 6 metabolites known be prognostically important in IPAH. Shown are the metabolite levels (*z* score) before and after exenatide infusion at each sampling site. SVC, superior vena cava; PA, pulmonary artery; RA, radial artery. *P < 0.05. **FDR-corrected *P* < 0.05. *P* values were determined by paired *t* test.

To explore mechanistic effects of exenatide, a targeted analysis was performed on metabolites previously shown to carry association with disease severity and prognosis of IPAH (16) and CTEPH (17). Six of 66 metabolites with prognostic value in IPAH changed acutely following exenatide infusion (Figure 3). Tricarboxylic acid (TCA) cycle intermediates malate and fumarate and free fatty acid hexadecadienoate, which are normally elevated in PAH and associated with poor prognosis, decreased in response to exenatide compared with baseline levels (Figure 3, A and B). Two amino acids with prognostic relevance in PAH, 1-methyl-4-imidazoleacetate (histidine metabolism) and vanillylmandelate (tyrosine metabolism), also reduced significantly following exenatide (Figure 3C). Exenatide resulted in a significant increase in levels of the free fatty acid, sphingomyelin (d18:2/14:0, d18:1/14:1).

**Figure 4:**
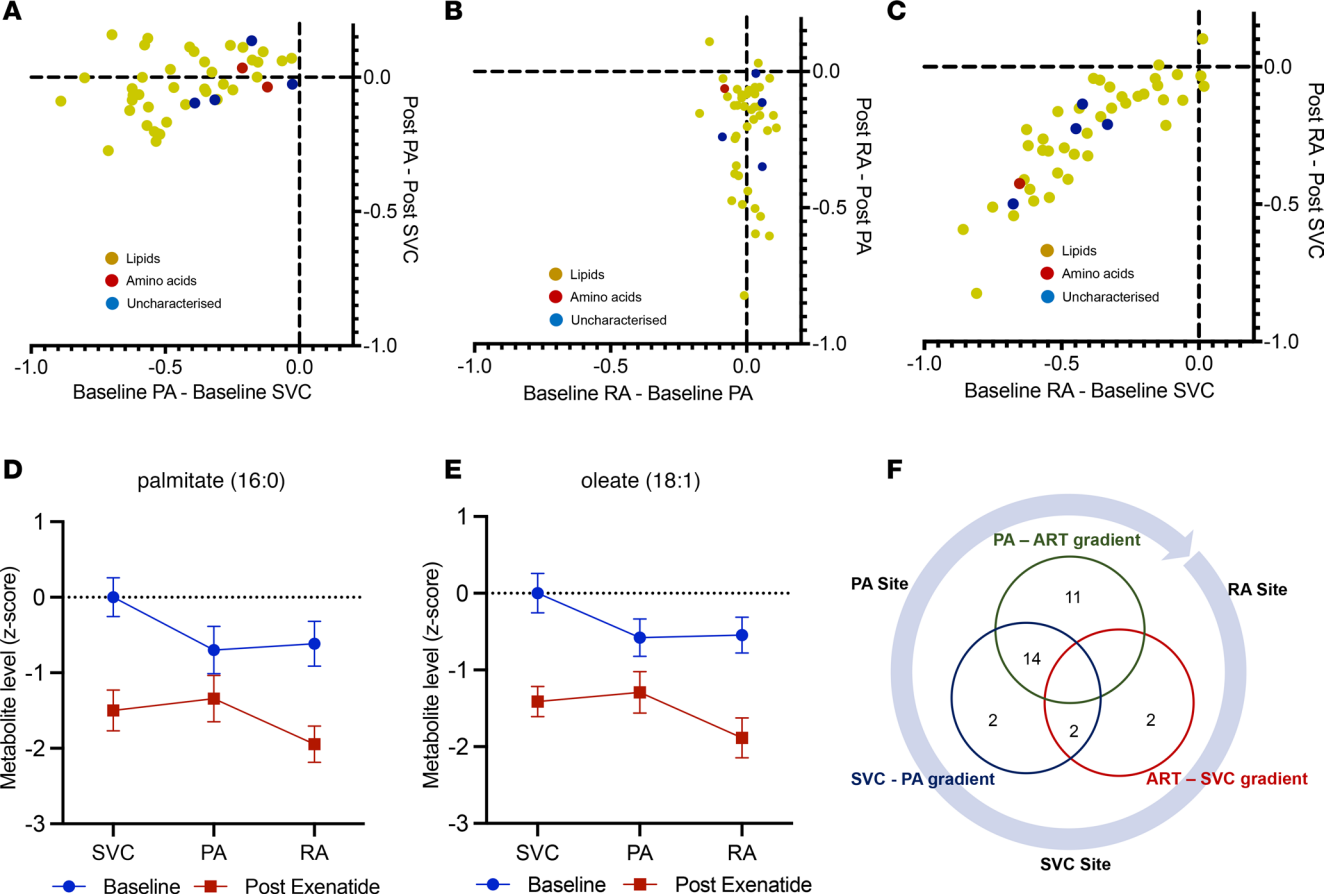
Change in metabolite gradients across sample sites before and after exenatide. (**A**–**C**) Shown are metabolite gradients between SVC-PA (**A**), PA-RA (**B**), RA-SVC (**C**) for 47 metabolites with significant change in level following exenatide infusion (FDR *q* < 0.05). (**D** and **E**) The metabolite levels at each sampling site for 2 free fatty acids — palmitate and oleate, respectively — known to be increased in PAH. Exenatide significantly reversed the direction of metabolite gradients across sampling sites. (**F**) The overlap in metabolite number demonstrating reversal of the direction of gradients with exenatide. Absolute difference in measurements reported (**A**–**C**). Data are shown as mean ± SEM (**D** and **E**).

We hypothesized that exenatide may have differing effects across different capillary beds. This concept was explored by analyzing the change in metabolite gradient across sampling sites (pulmonary artery [PA] to superior vena cava [SVC], radial artery [RA] to PA, and RA to SVC; Figure 4, A–C). Between the SVC and PA, exenatide reduced the metabolite gradient in the majority of metabolites and reversed the direction of the gradients in 16 metabolites (Figure 4A). Conversely, between the PA and systemic arterial circulation, exenatide increased the gradients in the majority of metabolites and reversed the gradient direction in 25 metabolites (Figure 4B), including 2 prognostically significant free fatty acids, palmitate (C16) and oleate (C18:1) (Figure 4, D and E).

### Preclinical component

CMR indices before and after exenatide infusion are summarized in Table 2. All rats had compensated RV systolic function defined as RV CO > 100 mL/min, at the time of the study. The monocrotaline (MCT) group demonstrated significantly higher RV end diastolic volume (EDV) index (*P* = 0.031), end systolic volume (ESV) index (*P* = 0.022), and RV mass index (*P* = 0.032), consistent with previous data supporting RV hypertrophy at this stage. Preinfusion baseline values of RV SVi and cardiac index (CI) were similar between the MCT and control groups. Exenatide infusion resulted in an overall 6% rise in heart rate in both groups compared with baseline (*P* < 0.01).

**Figure 5:**
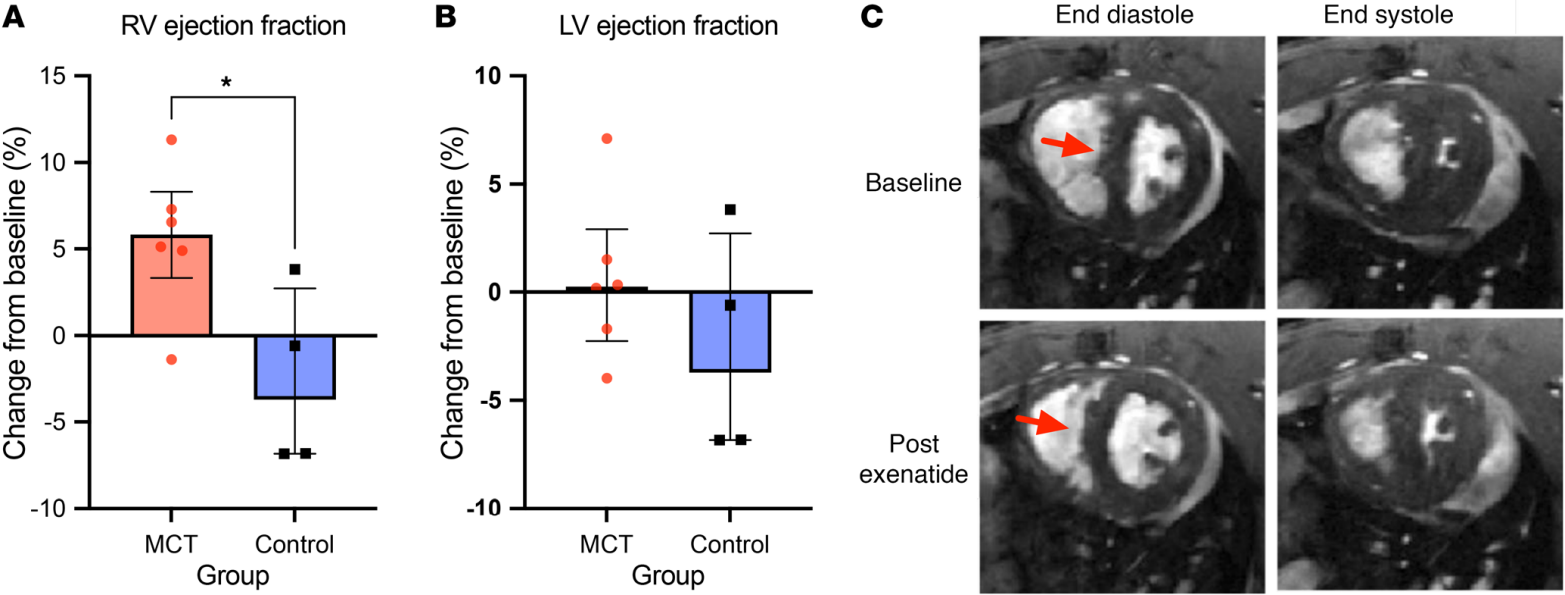
Effects of exenatide in the monocrotaline PAH model. (**A** and **B**) The changes in RV (**A**) and LV (**B**) ejection fraction from baseline following administration of exenatide in the MCT and control groups. (**C**) Representative original short-axis CMR images acquired at end diastole and end systole. Midventricular slices were obtained from cine images from MCT rats before and after exenatide infusion for 30 minutes. Arrows point to the interventricular septum displacement demonstrating reversal in septal flattening post exenatide. *P < 0.05. *P* values were determined by the independent sample Kruskal-Wallis test.

In the MCT group, exenatide resulted in lower RV EDV index (*P* = 0.027) and ESV index. There was also a reduction in intraventricular septum flattening of MCT rats in response to exenatide. RV ejection fraction (EF) increased (*P* = 0.046), with SVi and CI remaining unchanged. RV-to-PA coupling, as assessed by the indirect measurement of RV end systolic elastance/pulmonary arterial elastance (Ees/Ea), improved with exenatide. Left ventricular volumes and EF remained stable. No differences were observed in RV volumes and RV-to-PA coupling in the control rats. Changes in RV and LV EF in response to exenatide are shown in Figure 5, A and B. Representative CMR images from the study groups are shown in Figure 5C, with further delineation of changes in septal flattening shown in Supplemental Figure 3.

## Discussion

In 2 first-in-disease studies of 17 patients with IPAH or CTEPH, the i.v. GLP-1 agonist exenatide improved pulmonary vascular resistance, altered metabolites involved in myocardial fatty acid oxidation and oxidative phosphorylation, and — in a subset of patients undergoing pressure volume (PV) catheterization — improved RV-arterial coupling via an increase in RV elastance and reduction in afterload. Exenatide was well tolerated, demonstrating a nonsignificant reduction in plasma glucose, in keeping with its insulinomimetic effect. Mild nausea reported in 2 patients resolved on infusion termination. In both IPAH and CTEPH exenatide caused a rise in heart rate without development of arrhythmia, and patients with IPAH demonstrated improvements in SV and compliance. These results support an acute vasorelaxant effect of exenatide on the pulmonary vascular bed in a treated patient cohort with IPAH. In the rodent PAH model, significant improvement in CMR-derived indices of RV function was observed with exenatide infusion, characterized by a reversal in interventricular septal wall flattening and an increase in RV contractile function. These findings support a rationale for further exploration of the chronic therapeutic use of exenatide in PAH and CTEPH.

Reports of the acute effect of GLP-1 infusion on right heart function are limited with, to our knowledge, none to date including patients with IPAH or CTEPH, conditions that share a common pathophenotype of distal pulmonary vascular remodeling. Invasive hemodynamic studies of GLP-1 agonists have predominantly focused on left heart dysfunction and used either native GLP-1 peptide or synthetic GLP-1 agonists, such as exenatide. Clarke et al. administered native GLP-1 (7, 36) to patients with advanced heart failure, a proportion of whom exhibited increased PA pressure at baseline. They found a neutral hemodynamic effect of GLP-1 (7, 36), despite consistent perturbations in heart rate and glucose homeostasis (18). Furthermore, in a randomized crossover study of i.v. exenatide in patients with type 2 diabetes and congestive heart failure, no effect on PVR was demonstrated in those receiving the active drug (19).

Preclinical data on GLP-1 agonist effects in PAH are more encouraging, with several PAH animal models demonstrating a vasorelaxant effect of both native GLP-1 and GLP-1 agonists (9, 11, 20, 21). Mechanisms contributing to this effect include a reduction in plasma and lung tissue endothelin-1 and an increase in nitric oxide bioavailability — the latter mediated by phosphorylation and activation of endothelial nitric oxide synthase and soluble guanylate cyclase. In a PAH rodent model associated with systemic left-to-right shunting, exenatide also reduced PA pressure, restoring both vascular smooth muscle cell phenotype and vasoactive mediators, mitigating progression of pulmonary arteriopathy (22). GLP-1R is expressed on pulmonary vascular endothelium and smooth muscle cells, supporting the potential for GLP-1 agonists to modulate both nitiric oxide and endothelin pathways in PAH-associated vasoconstriction (12, 23). Over the time frame of acute infusion during the clinical study, acute reduction in PVR and improvement in PVC mediated by upregulation in the nitric oxide pathway is plausible, with less certain influence of alteration in endothelin signaling, which develops more slowly. Reduced circulating endogenous natriuretic peptides, commonly elevated in insulin-resistant states, have been suggested as an alternative mediator of pulmonary vasorelaxation, although their precise role and those of other circulating factors on the afterloaded RV and PAH lung vasculature requires further study (24).

In addition to pulmonary vasodilatation, both clinical and animal studies demonstrated acute improvements in RV contractility in response to exenatide. In a subset of patients with IPAH evaluated by PV catheterization, RV contractile recruitment following exenatide infusion restored RV-arterial coupling, a finding that was corroborated by increased RV contractile function in our PAH rodent model. These findings contrast with several prior LV studies that tested the hemodynamic effects of GLP-1 agonists in patients with reduced LV EF, in whom improvement in LV contractile responses has not been reliably demonstrated (25–30). In contrast and in support of our findings, atrial and ventricular trabeculae harvested from 72 patients with nonfailing myocardium exhibited a dose-dependent increase in cardiac contractility in response to exenatide mediated via the GLP-1R/cAMP/PKA pathway (31), which increases PKA-dependant phosphorylation of L-type Ca^2+^ channels, leading to improved cardiac contractility (32). RV volumetric changes also demonstrated a small increase in RV EDV in response to exenatide, and while only measured in a subset of 4 patients, this potentially represents a maladaptive response whereby improvement in SV is achieved through ventricular dilatation. Across all patients, exenatide led to an acute reduction in PVR, with increased CO driven predominantly by an increase in heart rate. As PVR reduction usually associates with a reduction in RV stroke work, an acute increase in the RV’s contractile response raises the potentially disadvantageous development of sympathetic overdrive, whereby greater volume loading and tachycardia offset the benefit toward the RV of pulmonary pressure reduction. Chronic studies are therefore required to understand the sustained effects of exenatide on RV stroke work and adaptation.

Among the milieu of neurohormonal activation, inflammation, and oxidative stress in PAH vasculature, the metabolic phenotype of the hypertrophied RV is characterized by suppression of fatty acid oxidation, upregulation in myocardial glycolysis, and increased capacity for glucose uptake (16, 17, 33, 34). The triggers, duration, and association with RV contractile performance of metabolic substrate shifts in PAH remain incompletely understood, although RV accumulation of long-chain fatty acids in PAH and association with cardiac steatosis and RV lipotoxicity is now recognized (35). Metabolites significantly downregulated following exenatide infusion predominated in pathways involving free fatty acid oxidation, a finding potentially supported by reversal in lipid gradients across the lung following exenatide administration. For example, hexadecanedioate, which lies in the fatty acid dicarboxylate pathway and was reduced acutely by exenatide, has been found to be elevated in patients with PAH compared with controls, reflecting a stress signal of dysfunctional lipid metabolism with adverse prognostic potential. In contrast, plasma sphingomyelins, the most abundant subclass of sphingolipids, are normally reduced in PAH and CTEPH, conferring a 2-fold increased mortality risk (16, 17). In our study, levels increased following exenatide; therefore, while chronic exenatide therapy in diabetes is typically associated with a reduction in sphingomyelins, evidence in PAH suggests that sphingomyelin pathways carry association with RV dilation and increased NT-proBNP (36). Mechanistically, an acute increase in plasma sphingomyelins could, therefore, be transient or, alternatively, represent an acute inhibitory effect on their enzymatic breakdown, halting their pathological conversion into proapoptotic ceramides, a key mediator of lipotoxic cardiac damage (35). Whether these changes could reflect a defense mechanism, potentially shielding the myocardium from lipotoxicity remains uncertain but underscores the possibility that exenatide may exert highly divergent, organ-specific effects.

A further potential consequence of downregulation in fatty acid oxidation mediated by exenatide across the lung may be the restoration of glucose oxidation in the mitochondrial matrix through competition for oxidative acetyl CoA production via the Randle cycle (37). Reduced mitochondrial RV fatty acid oxidation in PAH may develop even with adequate delivery of free fatty acids — for example, via suppression of TCA cycle intermediates (38). It is noteworthy, therefore, that 2 TCA cycle intermediates in particular, malate and fumarate, which are normally elevated in PAH (16, 39), were acutely decreased by exenatide (FDR *q* < 0.05). Acute suppression of TCA intermediates in response to exenatide could represent either improved mitochondrial efficiency or reduced oxidative flux. We noted no change in lactate and pyruvate on transpulmonary metabolite concentrations, which are both upstream substrates of the TCA cycle. While this potentially supports a shift toward a more efficient oxidative steady state following exenatide infusion, concomitant changes in CO as well as a contributory effect of left ventricular metabolites between pulmonary and RA sampling sites make true enhancement in mitochondrial cycle efficiency and glycolytic reversal in the pulmonary vasculature difficult to confirm.

There are several limitations to our study. Firstly, the sample sizes in both the clinical and preclinical components are small, with no replication of invasive hemodynamics in the animal model. In the clinical study, we were unable to include a sham (control) infusion arm design nor extend hemodynamic measurements beyond washout of exenatide, given that GLP-1R–mediated effects on endothelial NO synthase may take several hours to reverse. Instead, both exenatide dosing and infusion duration were kept constant, meaning it was administered at concentrations previously shown to be sufficient to reach a plasma concentration between 0.03 and 0.3 nmol/L within 15 minutes. Based on a previous pharmacokinetic study of exenatide (40), this resulted in an expected physiological increase in heart rate, in keeping with adequate systemic exenatide exposure. Finally, given known effects of exenatide on the liver and adipose tissue, we were unable to completely discriminate systemic effects of exenatide on plasma metabolomics from other organ beds from those derived from RV of lung tissue. To mitigate this, all patients were fasted for 6 hours prior to each procedure to minimize the confounding effects of nutritional intake, and all invasive studies were undertaken in the morning. Glucose levels before and after exenatide infusion were also not significantly different, making it unlikely that the mild hypoglycemic effect of exenatide could have accounted for metabolite level changes.

Despite contemporary advances in PAH therapies, there is an unmet need for treatments with potential pulmonary vasorelaxant properties that also modify the PAH disease course. Recent studies using novel pulmonary vasodilator therapies, including sotatercept, an Activin A ligand trap, are encouraging; however, many patients demonstrate persistently elevated PA pressures (5). Our study demonstrates an acute hemodynamic benefit of exenatide in IPAH, which is supported by improvements in CMR-derived indices of RV function in a PAH rodent model. These findings build on a growing evidence base of preclinical data for GLP-1 agonist use in pulmonary vascular disease.

## Methods

### Sex as a biological variable

Our study examined male and female animals, and similar findings are reported for both sexes. Sex was not included as a biological variable in the clinical study.

### Study design

**Figure 6:**
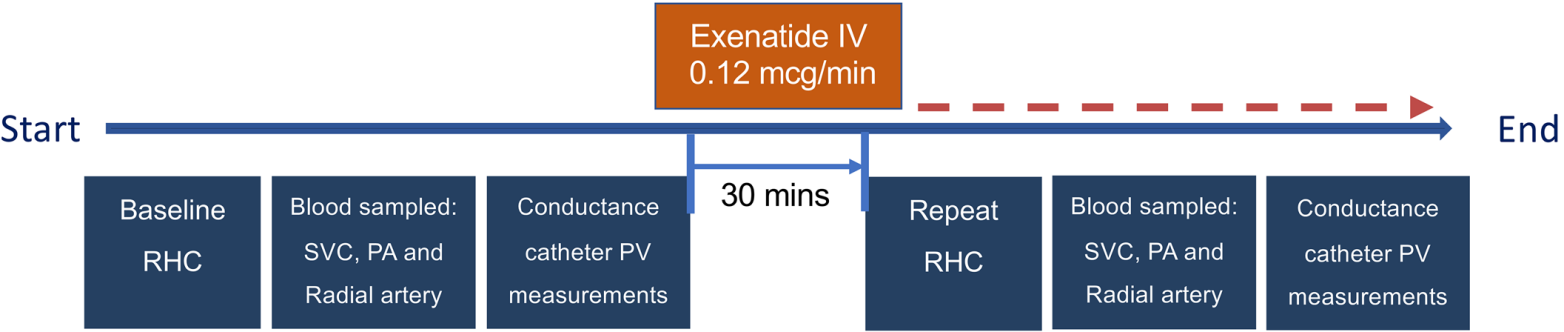
Study flowchart demonstrating sequence of measurements made at right heart catheterization before and after exenatide infusion. RHC, right heart catheterization; SVC, superior vena cava; PA, pulmonary artery; PV, pressure-volume; IV, intravenous.

Adult patients with a diagnosis of IPAH or CTEPH referred for routine clinical right heart catheterization (RHC) at the National Pulmonary Hypertension Service, Royal Brompton Hospital, were eligible. Exclusion criteria included renal impairment (eGFR < 60 mL/min/1.73 m^2^) and treatment with insulin or sulfonylureas. Figure 6 depicts the study flow.

### Exenatide infusion

Exenatide infusion was prepared in the cardiac catheterization laboratory immediately prior to administration, using methods previously published (41). First, 25 µg of exenatide (Byetta, AstraZeneca AB, Sweden) was added to a 250 mL bag of 0.9% saline. In total, 2.5 mL of human albumin (4%) were added to the bag to prevent binding of exenatide to infusion material. Infusions was delivered via a peripheral cannula with line filters, at a flow rate 72 mL/h (0.12 µg/min) for up to 40 minutes based on prior experience demonstrating stable exenatide plasma concentration after 15 minutes of infusion at this rate (40, 41).

### Safety and tolerability

Safety and tolerability were assessed using continuous vital sign and electrocardiography monitoring. Standard laboratory biochemical assessments were undertaken at the end of the study. Subjective tolerability of GLP-1 agonist infusion was evaluated by questioning the patients about adverse events, or by spontaneous reporting of adverse events. Patients were observed for at least 6 hours after completing the procedure and contacted the following day to assess for delayed adverse effects.

### Cardiac catheterization method

All patients were fasted for at least 6 hours prior to the cardiac catheterization, and the study assessments were performed in the morning with a prior 12-hour washout period from all other pulmonary vasodilatory substances. The study was performed in a quiet, environmentally controlled cardiac catheterization laboratory with an ambient temperature of 25°C. All study participants were nonsedated, and no other hemodynamically altering medications were given during the procedure. Baseline hemodynamics were obtained after a minimum of 30 minutes of rest in a supine position via insertion of a 6-Fr Swan-Ganz catheter which was advanced through a 7-Fr 10 cm sheath via the right basilic or internal jugular vein. Zero reference was set at mid-chest level. The catheter was advanced into the right atrium, RV, pulmonary arterial, and pulmonary capillary wedge positions under fluoroscopy. At each position, pressures were obtained at end-expiration averaged over several cardiac cycles. CO was obtained by Fick and thermodilution. CI was calculated as CO/body surface area. PVR was calculated as (mPAP − mPCWP)/CO.

Conductance catheterization of the RV was undertaken in a subset of patients following routine Swan-Ganz catheterization prior to start of the exenatide infusion. Prior to placement of the conductance catheter, blood resistivity was determined by rho cuvette. A 7-Fr high-fidelity conductance catheter (CD Leycom), was exchanged through the vascular sheath, and advanced under fluoroscopic guidance across the tricuspid valve toward the RV apex. Catheters were placed along the longitudinal axis of the ventricle to optimize the PV signal. Correct placement was confirmed fluoroscopically and by monitoring segmental volume phase relationships and counter-clockwise PV loop genesis.

Catheter calibration was performed according to the technique described by Baan et al. (42). Parallel conductance volume (Vc) was measured using the hypertonic saline injection technique through a side-channel port. At least 2 measurements of Vc were acquired using 5 mL 10% hypertonic saline, and the results were averaged. The coefficient α was calculated as the ratio between conductance catheter-derived CO and the reference CO from the Swan-Ganz study.

### Conductance catheter measurements

Conductance catheter data were analyzed offline. Conductance signals were calculated from dual-field excitation. Systolic function was assessed by RV stroke work index (RVSWI) and maximum rate of isovolumic pressure increase (dP/dt_max_). Diastolic function was assessed by maximum rate of isovolumic pressure decline (dP/dt_min_) and tau (τ), the time constant of isovolumic relaxation. τ represents the time constant of isovolumic pressure decay and is measured during active myocardial relaxation; it is calculated as a parameter in an exponential fit to the pressure channel data (Weiss’ method), using the following equation: P(t)= A × exp (−t/τ), where t is time and A is the fitted parameter. Compliance was calculated using the pulse pressure method: conductance-derived SV/PA pulse pressure. Single-beat estimation of Ees was carried out in all study participants using a sinusoidal curve fit algorithm written to estimate theoretical maximum isovolumic pressure from dP/dt_max_ and dP/dt_min_ during ventricular ejection (43). RV contractility (Ees) was taken as the gradient from theoretical maximal isovolumic pressure (P_max_) to the end-systolic point. RV afterload (Ea) was taken as the gradient from the end-systolic to end-diastolic points of the pressure-volume loop.

### Metabolomics analysis

Six blood samples were collected from each patient (102 samples total). Samples were collected from the SVC, PA, and RA before and after exenatide infusion to calculate pulmonary vascular metabolic flux across the pulmonary vascular bed (39). For each collection site, samples were drawn into 2 blood collection tubes — serum separating tube (SST) and ethylenediaminetetraacetic acid (EDTA). Each plasma and serum sample were processed as designated by the manufacturer’s specification. Briefly, for serum, collection tubes were inverted 5 times and then allowed to clot (left upright) for 30 minutes at room temperature (18°C–22°C). The SST tubes were centrifuged at 1,500*g* at room temperature (18°C–22°C) for 15 minutes. Plasma EDTA tubes were inverted 8–10 times immediately after collection and maintained at 4°C–8°C at all times following collection and during processing. EDTA tubes were centrifuged at 1,500*g* for 15 minutes at 4°C–8°C. Using separate disposable plastic Pasteur pipettes, plasma, and serum samples were aliquoted into FluidX tubes and stored at –80°C.

Nontargeted metabolomic profiling by ultraperformance liquid chromatography mass spectrometry was performed on the plasma samples using the Discovery HD4TM Global Metabolomics platform by Metabolon Inc. Data were provided as semiquantitative metabolite levels and comprised 1,347 biochemicals, 1,081 compounds of known identity (named biochemicals), and 266 compounds of unknown structural identity (unnamed biochemicals) annotated with pathways, as previously described (16).

### Preclinical component

Adult male Sprague-Dawley rats (body weight 200–230 g, Charles River) were used in the preclinical study. The rats were housed in 12-hour light/dark cycles with ad libitum access to water at standard laboratory conditions. For the analysis, sex was not considered as a biological variable.

The rats were randomly allocated to 2 groups: (a) control (*n* = 4) and (b) MCT-induced pulmonary hypertension (PH) group (MCT, *n* = 6). PH was induced by a s.c. injection of MCT solution (60 mg/kg). Cardiac magnetic resonance (CMR) was performed at an average of 17.5 days after injection in both groups.

#### CMR.

CMR imaging was conducted in the Biomedical Imaging Centre (BIC) at Imperial College, London (Hammersmith Campus). Images were acquired on a 9.4T Bruker scanner (Bruker BioSpec). The rats were anesthetized with isoflurane (5%) and kept/adjusted to maintain a respiratory rate of around 40–60 breaths per minute during the CMR. Heart rate was monitored by ECG throughout the CMR examination. Body temperature was monitored and kept at around 37°C using a heating mat.

All CMR acquisitions were prospectively triggered with the ECG R-wave and breathing rate. A T1-weighted gradient echo fast low angle shot (FLASH) sequence was used to acquire 2D multislice stack of images. The following parameters were used: repetition time (T_R_) was RR interval/number of frames (~6.2 ms for ~27 frames); echo time (TE) 2.3 ms; effective repetition time was the RR interval; flip angle was 18°; scan time ≤ 18 minutes. CINE image in-plane spatial resolution was (200 × 200) μm^2^ with a slice thickness of < 1.5 mm. Interslice thickness adjustments were made on a subject basis to cover both ventricles as well as to capture the following anatomical landmarks: the apex and base of the RV, the aortic valve and the top of LV wall.

#### GLP-1 infusion.

Exenatide (Byetta, AstraZeneca AB) was prepared to a final exenatide concentration of 2.5 μg/mL by adding to isotonic saline (0.9% NaCl with 1% BSA) and infusion prepared with exenatide concentration of 2.5 μg/mL. Albumin was added to prevent binding of exenatide to infusion lines (bovine serum albumin, Sigma-Aldrich, final concentration 0.1% BSA). The solution was administered as a continuous i.v. infusion at a rate of 0.25 μg/kg/min via an infusion pump (Harvard Apparatus). CMR acquisition was initiated 15 minutes after the start of the infusion, once the heart rate had stabilized. Cine scans were acquired before and after infusion for baseline and treatment comparison, heart rate (BPM), heart period (R-R interval length; ms), respiration (bpm), and temperature (°C) were noted during the scan process.

#### Image analysis.

The end-diastolic and end-systolic images were segmented manually by an experienced observer via a free open-source software ITK-Snap. Interventricular septum was considered as part of the LV wall. Trabeculae and papillary muscles were included into the blood pool volume for simplicity and applicability.

SV was calculated as the difference between end-diastolic volume (EDV) and end-systolic volume (ESV); SV = EDV – ESV. CO was derived from the equation: CO = HR × SV/1000, units are mL/min. The EF was assessed according to: EF = SV/EDV × 100%. Mass was calculated by multiplying the specific myocardial density (1.05 mg/mL) with the ED myocardial volume. All indexed parameters were matched to the BSA (44). This was calculated with Meeh’s formula: BSA = k × W^2/3^/10,000, where k is a constant 10 and W is body weight in grams, resulting BSA is in cm^2^ (45). The RV-to-PA coupling was determined by the RV SV/RV ESV ratio (46). To calculate the heart rate response during the challenge, the difference of average heart rate between pre and post scans was compared with baseline ([HR_post_ – Hr_pre_]/HR_pre_ × 100%).

### Statistics

For the hemodynamic and CMR data, continuous variables were summarized as mean ± SD or median and interquartile range (IQR). Between groups comparison was performed with the independent sample Kruskal-Wallis test, with post hoc pair-wise comparisons performed using the Mann-Whitney *U* test. Pre- and postintervention comparisons were performed using paired sample Wilcoxon signed-rank test. *P* < 0.05 was used to establish statistical significance.

We processed metabolite data as described previously (16). One patient in the IPAH group had a hemolyzed blood sample and was excluded from metabolomics data processing and analysis. One patient in the CTEPH group did not have a baseline SVC sample and was excluded from pair-wise analysis. A total of 94 samples were analyzed. Overall change in metabolite levels in response to GLP-1 agonism was assessed from blood sampled at the RA site. The metabolite levels were normalized by Box-Cox transformations (47), and samples where metabolites were undetected were imputed with the minimum detected level for the metabolite. Data in all sites were *z* score transformed on the basis of the mean and SD of pre-GLP1 infusion SVC samples for ease of comparisons. Paired 2-tailed *t* tests were used to identify metabolites that differed significantly between baseline and after GLP-1 infusion at each sample site. A targeted analysis of 66 known metabolites with prognostic significance in PAH and CTEPH was also performed (16, 17). Statistical analyses were performed using SPSS Statistics V27 (IBM Corp.), Excel (Microsoft), and R with RStudio and associated packages.

### Study approval

Informed written consent was obtained from all participants prior to study enrollment, and all study procedures were conducted in accordance with the Declaration of Helsinki. The study was approved by the local Human Research Ethics Committee (17/LO/1686). All animal experiments were conducted in accordance with the scientific procedures approved by the UK Home Office under Animals Act 1986.

### Data availability

Relevant available data on animal or clinical studies including metabolite data can be obtained directly from the corresponding author. Values for all data points in graphs are reported in the Supporting Data Values file.

## Author contributions

Conceptualization was contributed by CM and LZ. Methodology was contributed by CBS, MN, CJR, LZ, and CM. Investigation was contributed by CBS, MN, AK, LCP, NB, AA, KD, LZ, and CM. Visualization was contributed by CBS, CJR, LZ, and CM. Funding acquisition was contributed by MRW, LZ, and CM. Project administration was contributed by LZ and CM. Supervision was contributed by LZ and CM. Writing of the original draft was contributed by CBS, MN, LZ, CM. Review and editing were contributed by CBS, MG, LCP, KD, SW, CJR, LZ, and CM.

## Conflict of interest

The authors have declared that no conflict of interest exists.

## Funding support

Imperial College Biomedical Research Centre (BRC) funding support:

- Imperial College BRC funding award: Shadow Panel Grant number B0330/030 (LZ, CM).

## Supplementary Material

Supplemental data

Supporting data values

## Figures and Tables

**Table 1:**
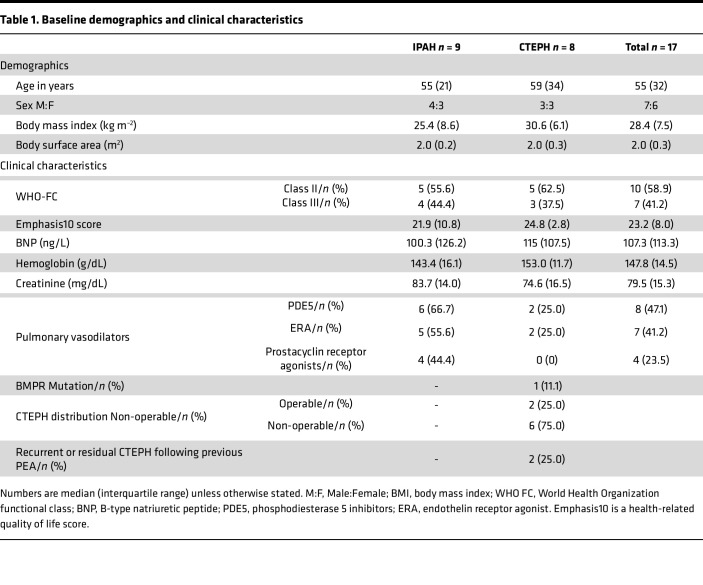
Baseline demographics and clinical characteristics

**Table 2:**
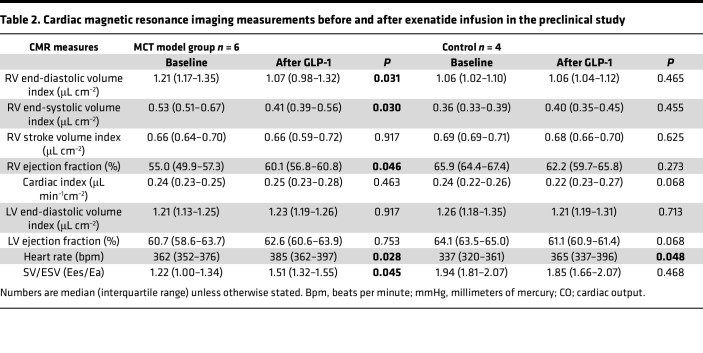
Cardiac magnetic resonance imaging measurements before and after exenatide infusion in the preclinical study

## Version 1 05/22/2026

Electronic publication

## References

[1] Humbert M (2014). Advances in therapeutic interventions for patients with pulmonary arterial hypertension. Circulation.

[2] Chang KY (2022). Mortality in pulmonary arterial hypertension in the modern era: early insights from the pulmonary hypertension association registry. J Am Heart Assoc.

[3] Farber HW (2015). Five-year outcomes of patients enrolled in the REVEAL registry. Chest.

[4] Hoeper MM (2022). Temporal trends in pulmonary arterial hypertension: results from the COMPERA registry. Eur Respir J.

[5] Hoeper MM (2023). Phase 3 Trial of Sotatercept for Treatment of Pulmonary Arterial Hypertension. N Engl J Med.

[6] Humbert M (2021). Sotatercept for the treatment of pulmonary arterial hypertension. N Engl J Med.

[7] Baggio LL (2018). GLP-1 receptor expression within the human heart. Endocrinology.

[8] Pyke C (2014). GLP-1 receptor localization in monkey and human tissue: novel distribution revealed with extensively validated monoclonal antibody. Endocrinology.

[9] Richter G (1993). GLP-1 stimulates secretion of macromolecules from airways and relaxes pulmonary artery. Am J Physiol.

[10] Honda J (2018). The glucagon-like peptide-1 receptor agonist liraglutide improves hypoxia-induced pulmonary hypertension in mice partly via normalization of reduced ET(B) receptor expression. Physiol Res.

[11] Wang J (2019). Glucagon-like peptide-1 (GLP-1) mediates the protective effects of dipeptidyl peptidase IV inhibition on pulmonary hypertension. J Biomed Sci.

[12] Ding L, Zhang J (2012). Glucagon-like peptide-1 activates endothelial nitric oxide synthase in human umbilical vein endothelial cells. Acta Pharmacol Sin.

[13] Bangshaab M (2019). Different mechanisms involved in liraglutide and glucagon-like peptide-1 vasodilatation in rat mesenteric small arteries. Br J Pharmacol.

[14] Ban K (2008). Cardioprotective and vasodilatory actions of glucagon-like peptide 1 receptor are mediated through both glucagon-like peptide 1 receptor-dependent and -independent pathways. Circulation.

[15] Brunner NW (2014). Impact of insulin resistance on ventricular function in pulmonary arterial hypertension. J Heart Lung Transplant.

[16] Rhodes CJ (2017). Plasma metabolomics implicates modified transfer RNAs and altered bioenergetics in the outcomes of pulmonary arterial hypertension. Circulation.

[17] Swietlik EM (2021). Plasma metabolomics exhibit response to therapy in chronic thromboembolic pulmonary hypertension. Eur Respir J.

[18] Clarke SJ (2019). Effects of acute GLP-1 infusion on pulmonary and systemic hemodynamics in patients with heart failure: a pilot study. Clin Ther.

[19] Nathanson D (2012). Effects of intravenous exenatide in type 2 diabetic patients with congestive heart failure: a double-blind, randomised controlled clinical trial of efficacy and safety. Diabetologia.

[20] Golpon HA (2001). Vasorelaxant effect of glucagon-like peptide-(7-36)amide and amylin on the pulmonary circulation of the rat. Regul Pept.

[21] Lee MY (2016). Liraglutide prevents and reverses monocrotaline-induced pulmonary arterial hypertension by suppressing ET-1 and enhancing eNOS/sGC/PKG pathways. Sci Rep.

[22] Roan JN (2018). Exendin-4 improves cardiovascular function and survival in flow-induced pulmonary hypertension. J Thorac Cardiovasc Surg.

[23] Barale C (2017). Glucagon-like peptide 1-related peptides increase nitric oxide effects to reduce platelet activation. Thromb Haemost.

[24] Kim M (2013). GLP-1 receptor activation and Epac2 link atrial natriuretic peptide secretion to control of blood pressure. Nat Med.

[25] Halbirk M (2010). Cardiovascular and metabolic effects of 48-h glucagon-like peptide-1 infusion in compensated chronic patients with heart failure. Am J Physiol Heart Circ Physiol.

[26] Munaf M (2012). A meta-analysis of the therapeutic effects of glucagon-like Peptide-1 agonist in heart failure. Int J Pept.

[27] Margulies KB (2016). Effects of liraglutide on clinical stability among patients with advanced heart failure and reduced ejection fraction: a randomized clinical trial. JAMA.

[28] Jorsal A (2017). Effect of liraglutide, a glucagon-like peptide-1 analogue, on left ventricular function in stable chronic heart failure patients with and without diabetes (LIVE)-a multicentre, double-blind, randomised, placebo-controlled trial. Eur J Heart Fail.

[29] Holman RR (2017). Effects of once-weekly exenatide on cardiovascular outcomes in type 2 diabetes. N Engl J Med.

[30] Sokos GG (2006). Glucagon-like peptide-1 infusion improves left ventricular ejection fraction and functional status in patients with chronic heart failure. J Card Fail.

[31] Wallner M (2015). Exenatide exerts a PKA-dependent positive inotropic effect in human atrial myocardium: GLP-1R mediated effects in human myocardium. J Mol Cell Cardiol.

[32] Drucker DJ (2018). Mechanisms of action and therapeutic application of glucagon-like peptide-1. Cell Metab.

[33] Piao L (2010). The inhibition of pyruvate dehydrogenase kinase improves impaired cardiac function and electrical remodeling in two models of right ventricular hypertrophy: resuscitating the hibernating right ventricle. J Mol Med (Berl).

[34] Zhang H (2017). Metabolic and proliferative state of vascular adventitial fibroblasts in pulmonary hypertension is regulated through a MicroRNA-124/PTBP1 (polypyrimidine tract binding protein 1)/pyruvate kinase muscle axis. Circulation.

[35] Brittain EL (2016). Fatty acid metabolic defects and right ventricular lipotoxicity in human pulmonary arterial hypertension. Circulation.

[36] Pi H (2023). Metabolomic signatures associated with pulmonary arterial hypertension outcomes. Circ Res.

[37] Al Batran R (2018). Glucagon-like peptide-1 receptor mediated control of cardiac energy metabolism. Peptides.

[38] Hemnes AR (2014). Evidence for right ventricular lipotoxicity in heritable pulmonary arterial hypertension. Am J Respir Crit Care Med.

[39] Lewis GD (2016). Metabolic profiling of right ventricular-pulmonary vascular function reveals circulating biomarkers of pulmonary hypertension. J Am Coll Cardiol.

[40] Lønborg J (2012). Exenatide reduces reperfusion injury in patients with ST-segment elevation myocardial infarction. Eur Heart J.

[41] Lønborg J (2012). Exenatide reduces final infarct size in patients with ST-segment-elevation myocardial infarction and short-duration of ischemia. Circ Cardiovasc Interv.

[42] Baan J (1984). Continuous measurement of left ventricular volume in animals and humans by conductance catheter. Circulation.

[43] McCabe C (2014). Right ventricular dysfunction in chronic thromboembolic obstruction of the pulmonary artery: a pressure-volume study using the conductance catheter. J Appl Physiol (1985).

[44] Sinning C (2018). Right ventricular index for risk stratification of patients with pulmonary arterial hypertension. Respiration.

[45] Gouma E (2012). A simple procedure for estimation of total body surface area and determination of a new value of Meeh’s constant in rats. Lab Anim.

[46] Vanderpool RR (2015). RV-pulmonary arterial coupling predicts outcome in patients referred for pulmonary hypertension. Heart.

[47] Box G, Cox D (1964). An analysis of transformations. J R Stat Soc Series B Stat Methodol.

