# Supplementary material for: Hemodynamic and metabolomic responses to infusion of GLP-1 agonist exenatide in pulmonary arterial hypertension
Source: JCI Insight. 2026 May 22;11(10):e202660. doi: 10.1172/jci.insight.202660 (PMC13232712; doi:10.1172/jci.insight.202660)
Supplement: Supplemental data [file jciinsight-11-202660-s274.pdf]

LIST OF SUPPLEMENTARY MATERIALS

Figure S1: Change in blood glucose level with administration of GLP-1 agonist exenatide

Figure S2. Change in metabolites with GLP-1 agonist Exenatide infusion across the heart and lungs.

Figure S3. Representative images illustrating the septal shift at end-systole (dotted lines) for all scanned MCT rats (R1-6).

Table S1: Changes in haemodynamics pre and post GLP-1 infusion.

Table S2. Individual patients' haemodynamic measurements pre and post infusion of GLP-1 agonist Exenatide.

Table S3: Changes in right ventricular performance pre and post GLP-1 infusion

COMPETING INTERESTS

None of the authors have any conflicts of interest relevant to this study.

Supplementary Material

Figure S1

Change in blood glucose level with administration of GLP-1 agonist exenatide. Changes in plasma glucose pre and post exenatide infusion in the included clinical study patients.

Figure S2

Change in metabolites with GLP-1 agonist Exenatide infusion across the heart and lungs. Shown are the mean change in metabolite levels at radial arterial site following exenatide infusion compared to baseline. Red dots are metabolites with significant change with false discovery rate (FDRq < 0.05), blue dots are metabolites with a significant change ($p < 0.05$), and black dots are metabolites with no significant difference. Negative values represent a reduction in the metabolite level following exenatide infusion. Selected metabolites with known prognostic significance in idiopathic PAH are named. Detailed values for mean change in metabolite level are provided in Table S4.

Figure S3

Representative images illustrating the septal shift at end-systole (dotted lines) for all scanned MCT rats (R1-6). Dotted lines outline the septal shift pre and post infusion of exenatide.

Supplementary Material

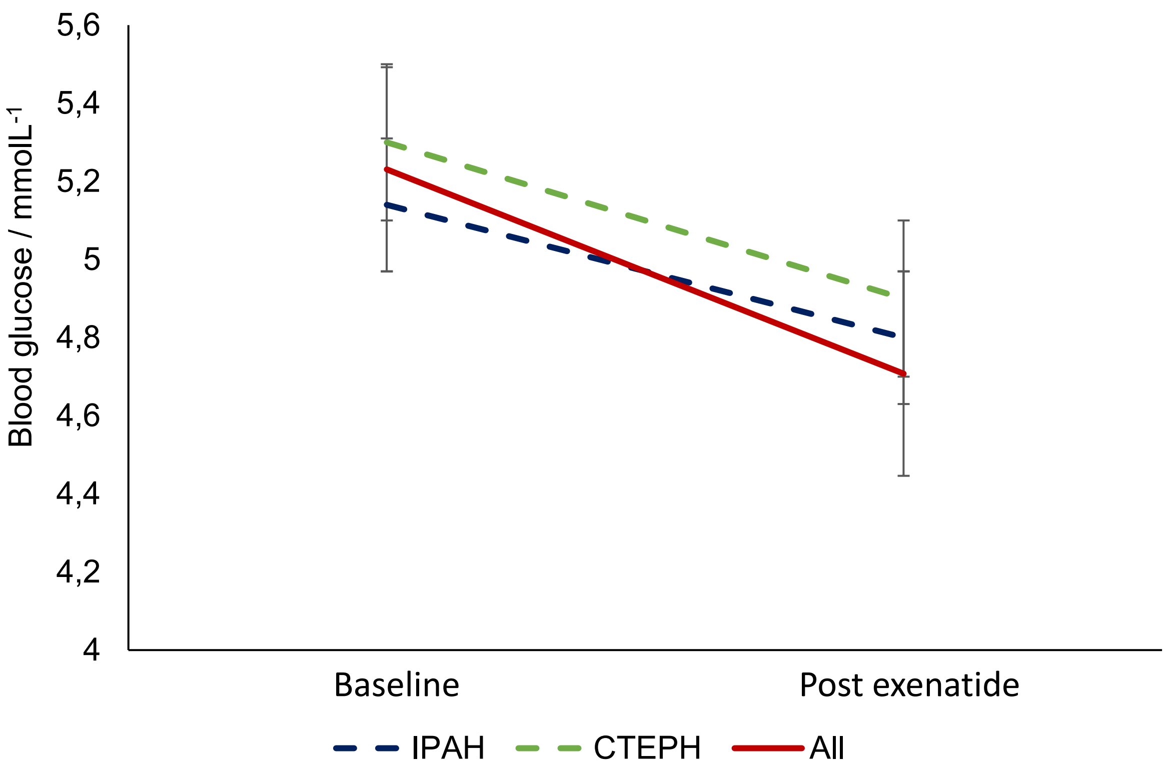

Figure S1

Changes in plasma glucose pre and post exenatide infusion in the included clinical study patients.

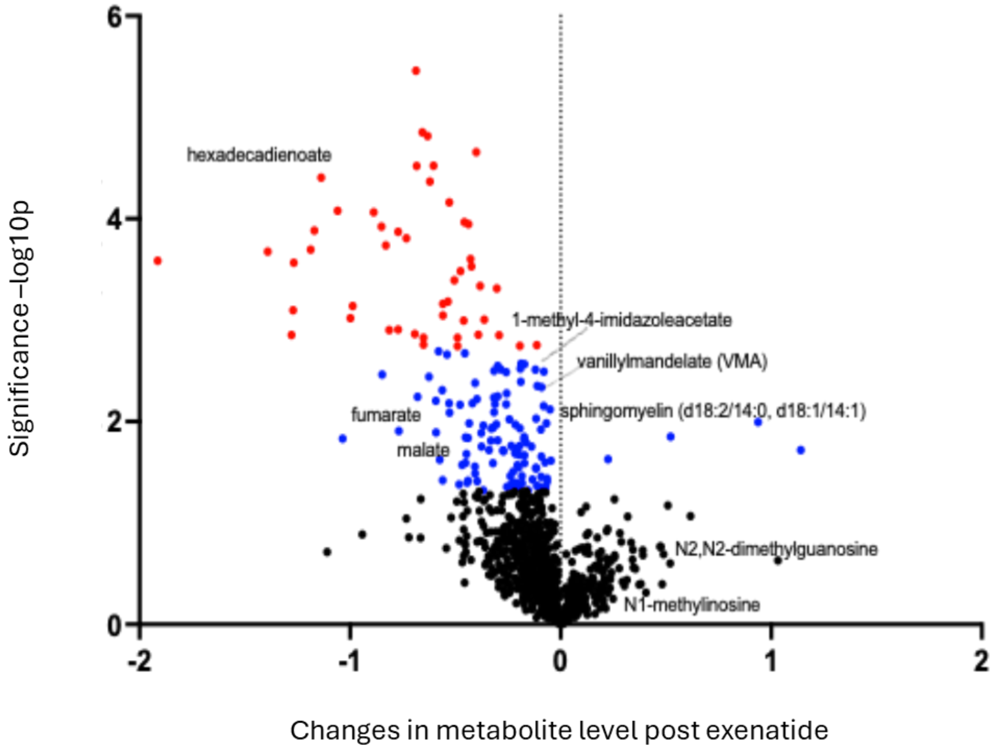

Figure S2

Change in metabolites following exenatide infusion across the heart and lungs. Shown are the mean change in metabolite levels at radial arterial site following exenatide infusion compared to baseline. Red dots are metabolites with significant change with false discovery rate (FDRq < 0.05), blue dots are metabolites with a significant change ($p < 0.05$), and black dots are metabolites with no significant difference. Negative values represent a reduction in the metabolite level following exenatide infusion. Selected metabolites with known prognostic significance in idiopathic PAH are named. Detailed values for mean change in metabolite level are provided in Table S4.

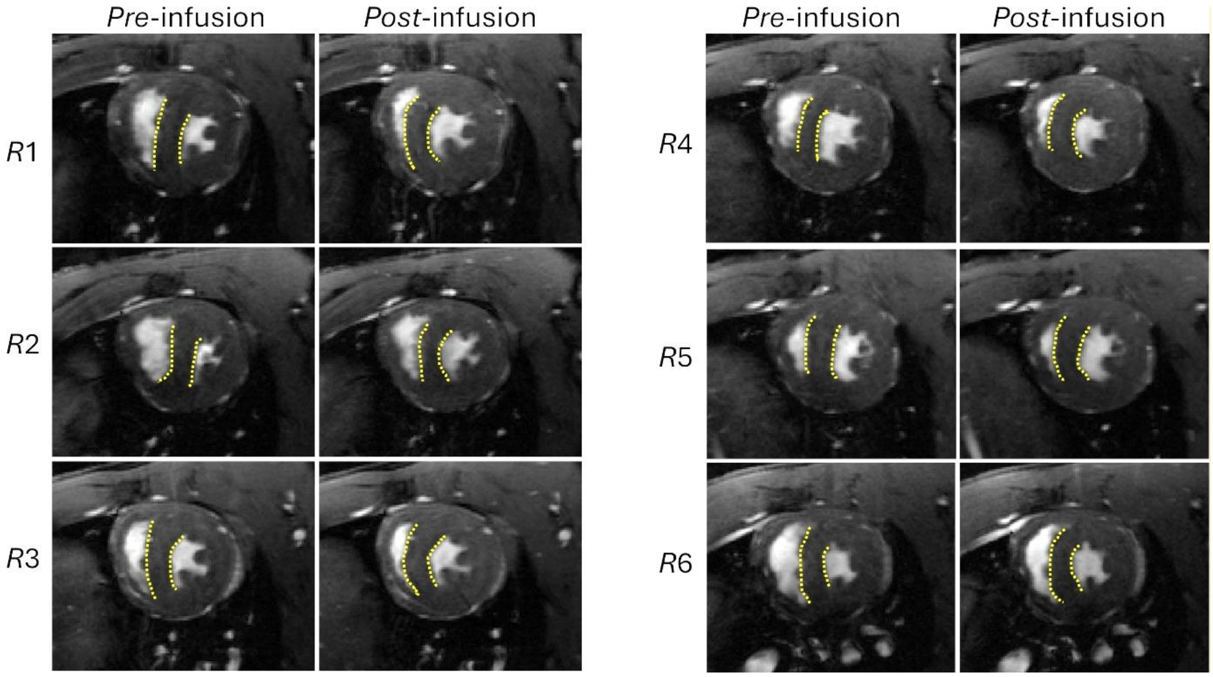

Figure S3

Representative images illustrating the septal shift at end-systole (dotted lines) for all scanned MCT rats (R1-6). Dotted lines outline the septal shift pre and post infusion of exenatide.

**Haemodynamics**	**IPAH group N = 9**			**CTEPH group N = 8**			**Total cohort N = 17**		
	**Baseline**	**Post exenatide**	**P**	**Baseline**	**Post exenatide**	**P**	**Baseline**	**Post exenatide**	**P**
HR (bpm)	73 (21)	79 (9)	**0.005**	78 (18)	88 (8)	**0.005**	75 (16)	84 (14)	**<0.001**
SBP (mmHg)	109 (12)	112 (24)	0.95	130 (14)	132 (18)	0.19	126 (24)	126 (18)	0.61
DBP (mmHg)	65 (5)	71 (10)	0.85	84 (7)	82 (12)	0.25	75 (21)	75 (17)	0.58
sMAP (mmHg)	78 (7)	89 (10)	0.82	95 (4)	96 (10)	0.34	86 (18)	88 (16)	0.55
Arterial O_2 saturation (%)	95 (5)	96 (7)	0.88	95 (2)	94 (3)	0.53	95 (4)	95 (4)	0.66
mRAP (mmHg)	5 (7)	5 (4)	0.56	6 (5)	7 (5)	0.611	5 (6)	6 (5)	0.41
mPAP (mmHg)	45 (11)	40 (11)	**0.043**	46 (18)	41 (19)	**0.008**	45 (15)	40 (18)	**0.001**
PCWP (mmHg)	6 (4)	7 (4)	0.59	10 (5)	11 (5)	0.127	8 (7)	9 (4)	0.17
PA O_2 saturation (%)	71 (5)	76 (5)	**0.005**	66 (12)	69 (12)	**0.039**	68 (10)	72 (13)	**0.012**
SV (ml)	68 (20)	70 (19)	0.13	45 (16)	45 (13)	0.74	55 (29)	58 (26)	0.17
CO (L/min)	4.4 (1.8)	5.7 (1.5)	**0.004**	3.5 (0.8)	4.3 (0.9)	**0.004**	3.9 (1.5)	4.8 (2.1)	**<0.001**
CI (L/min/m^2)	2.2 (0.1)	2.7 (0.5)	**0.005**	1.8 (0.5)	1.9 (0.5)	**0.005**	2.1 (0.6)	2.4 (0.9)	**0.004**
PVR (Woods units)	6.8 (7.8)	4.9 (4.5)	**0.018**	9.4 (8.4)	7.0 (7.4)	**0.001**	7.8 (8.0)	5.9 (5.0)	**<0.001**
PVC (ml/mmHg)	1.3 (1.0)	1.6 (0.9)	**0.043**	0.9 (0.8)	1.1 (1.0)	0.058	1.1 (1.0)	1.3 (1.1)	**0.006**

Table S1

Changes in haemodynamics pre and post exenatide infusion. Numbers are median (interquartile range) unless otherwise stated. IPAH: idiopathic pulmonary arterial hypertension; CTEPH: chronic thromboembolic pulmonary arterial hypertension; HR: heart rate; bpm: beats per minute; SBP: systolic blood pressure; DBP: diastolic blood pressure, sMAP: systemic mean arterial pressure; O_2: oxygen; mRAP: mean right atrial pressure; mPAP: mean pulmonary artery pressure; PCWP: mean pulmonary capillary wedge pressure; PA: pulmonary artery; SV: stroke volume; CO: cardiac output; CI: cardiac index; PVR: pulmonary vascular resistance; PVC: pulmonary vascular compliance.

Patient No	Diagnosis	Disease specifics	CTEPH distribution	mPAP-pre (mmHg)	mPAP-post (mmHg)	PCWP-pre (mmHg)	PCWP-post (mmHg)	PASat-pre (mmHg)	PASat-post (mmHg)	SV-pre (ml)	SV-post (ml)	CO-pre (L/ min)	CO-post (L/ min)	PVR-pre (WU)	PVR-post (WU)
**1**	IPAH	BMPR2	-	47	48	8	9	74.8	76.1	73	71	6.39	6.63	6.1	5.9
**2**	IPAH	-	-	45	41	3	3	71.2	76.5	55	63	3.03	4.52	13.9	8.4
**3**	IPAH	-	-	26	24	8	7	70.2	72.0	77	81	4.54	5.70	4.0	3.0
**4**	IPAH	-	-	43	40	4	7	71.3	72.6	82	83	6.19	6.78	6.3	4.9
**5**	IPAH	-	-	33	25	9	11	58.8	61.9	68	69	4.99	5.36	4.8	2.6
**6**	IPAH	Positive NO vasorecativity	-	45	30	3	1	76.6	80.9	53	48	2.69	3.38	15.6	8.9
**7**	IPAH	BMPR2	-	54	50	8	9	61.1	62.5	39	44	3.23	3.87	14.2	10.6
**8**	IPAH	-	-	36	32	6	8	69.7	76.4	75	86	4.52	6.02	6.6	3.9
**9**	IPAH	-	-	50	41	13	12	95.0	96.0	59	66	4.73	5.92	7.8	4.9
**10**	CTEPH	-	Operable	45	40	13	13	65.7	67.9	45	48	3.39	4.06	9.4	6.7
**11**	CTEPH	-	Non-operable	47	43	11	11	71.0	73.1	57	58	3.86	4.41	9.3	7.3
**12**	CTEPH	-	Non-operable	30	28	8	9	68.8	71.6	44	46	3.69	4.22	6.0	4.5
**13**	CTEPH	-	Non-operable	55	49	12	13	54.0	55.0	33	34	2.90	3.13	14.8	11.5
**14**	CTEPH	-	Non-operable	48	46	3	5	57.9	60.1	39	40	3.23	3.49	13.9	11.7
**15**	CTEPH	-	Operable	33	24	3	4	70.0	70.8	75	77	6.02	6.23	5.0	3.2
**16**	CTEPH	-	Non-operable	25	23	12	13	68.8	69.6	53	44	4.06	3.88	3.2	2.6
**17**	CTEPH	-	Non-operable	69	64	11	11	42.3	44.5	37	34	2.80	3.10	20.7	17.1

Table S2

Individual patients' haemodynamic measurements pre and post exenatide. IPAH: idiopathic pulmonary arterial hypertension; CTEPH: chronic thromboembolic pulmonary hypertension; mPAP: mean pulmonary artery pressure; PCWP: pulmonary capillary wedge pressure; PASat: pulmonary artery oxygen saturation; CO: cardiac output; PVR: pulmonary vascular resistance in Wood Units; NO: nitric oxide.

	Baseline	Post exenatide
**RV volumes**		
Stroke volume index (ml beat^{-1}m^{-2})	33.5 (4.2)	37.1 (5.0) *
End-diastolic volume index (ml m^{-2})	73.5 (7.6)	75.5 (7.1) *
End-systolic volume index (ml m^{-2})	40.8 (7.1)	38.1 (7.1) *
**RV systolic function**		
dP/dt max (mmHg s^{-1})	480.0 (124.3)	555.0 (191.5)
E_{ES} (mmHg ml^{-1})	0.51 (0.09)	0.65 (0.11) *
RV ejection fraction (%)	42.6 (4.1)	46.1 (3.8) *
Stroke Work (mmHg ml)	3305.5 (431.0)	3705.5 (274.0) *
**RV afterload**		
E_A (mmHg ml^{-1})	0.95 (0.63)	0.83 (0.56) *
**RV pulmonary arterial coupling**		
Ees/Ea	0.65 (0.21)	0.97 (0.46) *
**RV diastolic function**		
Tau (ms)	40.0 (1.5)	41.0 (3.0)
dP/dt min (mmHg s^{-1})	- 521.5 (143.5)	- 550.0 (167.5)
End-diastolic pressure (mmHg)	8.5 (4.3)	8.0 (4.8)

Table S3

Changes in right ventricular performance pre and post exenatide infusion in a subset of PAH patients (n=4). Numbers are median (interquartile range) unless otherwise stated. ml: millilitres; l: litres; RV: right ventricle; E_{ES}: Ventricular end-systolic elastance; E_A: pulmonary arterial elastance. * denotes statistically significant difference ($p < 0.05$) on a pair-wise comparison between pre and post measurements.

Pathway	Metabolite name	Mean difference	95% CI		P	FDR (Q)
			LL	UL		
Amino Acid	xanthurenate	-0.36	-0.55	-0.17	0.00	0.03
Amino Acid	kynurenate	-0.39	-0.60	-0.18	0.00	0.04
Amino Acid	vanillylmandelate (VMA)	-0.32	-0.53	-0.11	0.01	0.10
Amino Acid	dopamine 3-O-sulfate	-0.08	-0.14	-0.03	0.01	0.11
Amino Acid	1-methyl-4-imidazoleacetate	-0.30	-0.53	-0.07	0.02	0.18
Amino Acid	gentisate	-0.20	-0.36	-0.03	0.02	0.22
Amino Acid	N-acetylkynurenine (2)	0.22	0.03	0.41	0.02	0.23
Amino Acid	methylsuccinate	-0.32	-0.60	-0.04	0.03	0.24
Amino Acid	8-methoxykynurenate	-0.17	-0.32	-0.02	0.03	0.24
Amino Acid	3-methylhistidine	-0.09	-0.18	-0.01	0.03	0.30
Amino Acid	N-lactoyl phenylalanine	-0.21	-0.41	-0.01	0.04	0.32
Amino Acid	leucine	-0.37	-0.73	0.00	0.05	0.34
Amino Acid	thyroxine	-0.22	-0.44	0.00	0.05	0.35
Amino Acid	alanine	-0.34	-0.68	0.01	0.05	0.35
Amino Acid	N-formylphenylalanine	-0.36	-0.74	0.02	0.06	0.37
Amino Acid	isovalerate (i5:0)	-0.49	-1.01	0.03	0.06	0.38
Amino Acid	N-acetylphenylalanine	-0.16	-0.33	0.01	0.07	0.39
Amino Acid	N-acetyltaurine	-0.30	-0.64	0.03	0.08	0.40
Amino Acid	taurine	-0.25	-0.53	0.04	0.09	0.43
Amino Acid	N-acetylcitrulline	-0.52	-1.13	0.09	0.09	0.44
Amino Acid	isovalerylglycine	-0.38	-0.83	0.08	0.10	0.46
Amino Acid	N-lactoyl isoleucine	-0.19	-0.42	0.04	0.10	0.46
Amino Acid	4-hydroxyglutamate	-0.17	-0.38	0.04	0.10	0.47
Amino Acid	N-formylanthranilic acid	-0.20	-0.45	0.05	0.10	0.47
Amino Acid	ethylmalonate	-0.11	-0.26	0.03	0.11	0.48
Amino Acid	cysteine	-0.46	-1.05	0.13	0.12	0.49
Amino Acid	tyrosine	-0.31	-0.71	0.09	0.12	0.51

Amino Acid	isoleucine	-0.27	-0.63	0.09	0.13	0.51
Amino Acid	indolepropionate	-0.06	-0.14	0.02	0.13	0.51
Amino Acid	S-methylmethionine	-0.05	-0.11	0.02	0.13	0.52
Amino Acid	2-oxoarginine*	-0.10	-0.22	0.03	0.13	0.52
Amino Acid	fructosyllysine	-0.45	-1.05	0.16	0.14	0.52
Amino Acid	6-bromotryptophan	0.17	-0.06	0.41	0.14	0.52
Amino Acid	gamma-carboxyglutamate	-0.66	-1.58	0.25	0.14	0.52
Amino Acid	6-oxopiperidine-2-carboxylate	-0.25	-0.59	0.10	0.14	0.53
Amino Acid	N-lactoyl leucine	-0.28	-0.66	0.11	0.15	0.53
Amino Acid	N-acetylisoleucine	-0.38	-0.91	0.15	0.15	0.54
Amino Acid	methionine sulfoxide	-0.29	-0.69	0.12	0.15	0.54
Amino Acid	succinoyltaurine	-0.15	-0.36	0.06	0.15	0.54
Amino Acid	N-acetyltyrosine	-0.17	-0.42	0.07	0.15	0.54
Amino Acid	imidazole lactate	-0.21	-0.52	0.09	0.16	0.55
Amino Acid	beta-hydroxyisovalerate	0.47	-0.23	1.17	0.17	0.56
Amino Acid	phenol sulfate	-0.03	-0.06	0.01	0.17	0.56
Amino Acid	N-acetylglutamine	-0.14	-0.34	0.07	0.17	0.56
Amino Acid	N-acetylhistidine	-0.12	-0.30	0.06	0.18	0.56
Amino Acid	2-methylserine	-0.54	-1.36	0.28	0.18	0.56
Amino Acid	serine	-0.22	-0.54	0.11	0.18	0.56
Amino Acid	creatinine	-0.19	-0.47	0.10	0.18	0.56
Amino Acid	tryptophan betaine	-0.06	-0.16	0.03	0.18	0.56
Amino Acid	aspartate	-0.17	-0.43	0.09	0.18	0.56
Amino Acid	2,3-dihydroxy-2-methylbutyrate	0.34	-0.18	0.87	0.18	0.56
Amino Acid	N6,N6-dimethyllysine	-0.10	-0.24	0.05	0.18	0.56
Amino Acid	N-lactoyl valine	-0.17	-0.42	0.09	0.19	0.56
Amino Acid	dimethylglycine	-0.08	-0.21	0.05	0.19	0.56
Amino Acid	asparagine	-0.28	-0.72	0.16	0.19	0.56
Amino Acid	3-methylglutaconate	-0.23	-0.58	0.13	0.19	0.56
Amino Acid	N,N-dimethylalanine	0.19	-0.11	0.49	0.19	0.56

Amino Acid	trans-urocanate	-1.11	-2.85	0.63	0.19	0.56
Amino Acid	guanidinoacetate	-0.18	-0.46	0.10	0.20	0.56
Amino Acid	threonine	-0.13	-0.34	0.08	0.20	0.56
Amino Acid	4-methyl-2-oxopentanoate	-0.17	-0.43	0.10	0.20	0.56
Amino Acid	isovalerylcarnitine (C5)	-0.17	-0.43	0.10	0.20	0.56
Amino Acid	cystine	0.13	-0.08	0.35	0.20	0.57
Amino Acid	N-acetyl-2-aminoadipate	-0.28	-0.72	0.16	0.20	0.57
Amino Acid	3-hydroxy-2-ethylpropionate	-0.22	-0.57	0.14	0.22	0.59
Amino Acid	tryptophan	0.26	-0.17	0.68	0.22	0.59
Amino Acid	cysteine s-sulfate	0.22	-0.15	0.58	0.22	0.60
Amino Acid	N-acetylaspartate (NAA)	-0.25	-0.68	0.17	0.22	0.60
Amino Acid	N-acetylserine	-0.14	-0.38	0.10	0.23	0.60
Amino Acid	formiminoglutamate	-0.07	-0.18	0.05	0.23	0.60
Amino Acid	hydantoin-5-propionate	-0.06	-0.16	0.04	0.23	0.60
Amino Acid	N2-acetyl,N6-methyllysine	0.05	-0.04	0.15	0.24	0.61
Amino Acid	N-acetylthreonine	-0.47	-1.29	0.36	0.24	0.61
Amino Acid	N-delta-acetylornithine	-0.06	-0.15	0.04	0.25	0.61
Amino Acid	3-amino-2-piperidone	-0.10	-0.28	0.08	0.25	0.61
Amino Acid	histidine	-0.18	-0.51	0.15	0.26	0.62
Amino Acid	N6-acetyllysine	-0.25	-0.71	0.21	0.26	0.62
Amino Acid	beta-citrylglutamate	-0.36	-1.01	0.30	0.26	0.62
Amino Acid	phenylalanine	-0.28	-0.80	0.24	0.27	0.63
Amino Acid	N-methylproline	-0.04	-0.11	0.03	0.27	0.63
Amino Acid	arginine	-0.13	-0.39	0.12	0.28	0.64
Amino Acid	N-acetylcarnosine	-0.16	-0.46	0.14	0.28	0.64
Amino Acid	alpha-hydroxyisovalerate	-0.08	-0.24	0.07	0.28	0.64
Amino Acid	alpha-ketobutyrate	0.17	-0.16	0.51	0.29	0.64
Amino Acid	isobutyrylglycine	0.21	-0.19	0.60	0.29	0.64
Amino Acid	1-ribosyl-imidazoleacetate*	-0.14	-0.41	0.13	0.29	0.64
Amino Acid	N-acetylarginine	-0.10	-0.28	0.09	0.29	0.64

Amino Acid	ornithine	-0.13	-0.37	0.12	0.29	0.64
Amino Acid	4-guanidinobutanoate	-0.13	-0.39	0.13	0.30	0.65
Amino Acid	alpha-ketoglutaramate*	-0.13	-0.40	0.14	0.31	0.65
Amino Acid	m-tyramine sulfate	-0.27	-0.81	0.28	0.31	0.66
Amino Acid	oxindolylalanine	-0.32	-0.98	0.34	0.32	0.66
Amino Acid	indoleacetate	-0.07	-0.22	0.08	0.32	0.67
Amino Acid	hypotaurine	-0.16	-0.48	0.17	0.32	0.67
Amino Acid	tiglylcarnitine (C5:1-DC)	-0.09	-0.27	0.09	0.32	0.67
Amino Acid	1-methyl-5-imidazoleacetate	-0.06	-0.17	0.06	0.33	0.67
Amino Acid	S-methylcysteine sulfoxide	-0.04	-0.12	0.04	0.33	0.67
Amino Acid	methionine	-0.24	-0.76	0.27	0.33	0.67
Amino Acid	N-lactoyl tyrosine	-0.07	-0.21	0.08	0.33	0.67
Amino Acid	vanillactate	-0.06	-0.21	0.08	0.34	0.68
Amino Acid	2-hydroxy-3-methylvalerate	-0.26	-0.82	0.31	0.35	0.69
Amino Acid	1-methyl-5-imidazolelactate	-0.07	-0.22	0.09	0.36	0.69
Amino Acid	hydroxy-N6,N6,N6-trimethyllysine*	0.22	-0.28	0.72	0.36	0.70
Amino Acid	2-aminobutyrate	-0.09	-0.32	0.13	0.39	0.70
Amino Acid	3-hydroxyisobutyrate	-0.14	-0.47	0.19	0.39	0.70
Amino Acid	cysteinylglycine disulfide*	-0.14	-0.48	0.20	0.39	0.70
Amino Acid	2,3-dihydroxy-5-methylthio-4-pentenoate (DMTPA)*	-0.14	-0.49	0.20	0.40	0.70
Amino Acid	indolelactate	-0.08	-0.27	0.11	0.40	0.70
Amino Acid	S-1-pyrroline-5-carboxylate	-0.24	-0.84	0.36	0.40	0.71
Amino Acid	3-methoxytyramine sulfate	-0.09	-0.32	0.14	0.41	0.71
Amino Acid	N-succinyl-phenylalanine	0.08	-0.13	0.30	0.41	0.71
Amino Acid	cys-gly, oxidized	-0.12	-0.43	0.19	0.42	0.71
Amino Acid	N-carbamoylvaline	0.15	-0.23	0.52	0.42	0.71
Amino Acid	N-acetyl-3-methylhistidine*	-0.06	-0.22	0.10	0.42	0.72
Amino Acid	N-acetyl-isoputreanine	0.13	-0.20	0.46	0.42	0.72
Amino Acid	proline	-0.16	-0.58	0.26	0.43	0.73
Amino Acid	3-methoxytyrosine	-0.06	-0.22	0.10	0.43	0.73

Amino Acid	4-methoxyphenol sulfate	0.07	-0.12	0.26	0.44	0.73
Amino Acid	1-methylhistidine	0.11	-0.19	0.42	0.45	0.73
Amino Acid	cysteine-glutathione disulfide	-0.19	-0.73	0.34	0.45	0.73
Amino Acid	N-acetylalanine	-0.14	-0.53	0.25	0.45	0.73
Amino Acid	citrulline	-0.15	-0.56	0.26	0.46	0.73
Amino Acid	lanthionine	0.19	-0.34	0.71	0.46	0.74
Amino Acid	N6-methyllysine	-0.03	-0.10	0.05	0.46	0.74
Amino Acid	3-methyl-2-oxobutyrate	-0.12	-0.45	0.22	0.47	0.74
Amino Acid	indoleacetylglutamine	-0.05	-0.20	0.10	0.48	0.75
Amino Acid	4-hydroxyphenylacetate	-0.14	-0.56	0.28	0.48	0.75
Amino Acid	sarcosine	0.40	-0.80	1.61	0.49	0.75
Amino Acid	argininate*	-0.05	-0.22	0.11	0.50	0.76
Amino Acid	5-(galactosylhydroxy)-lysine	-0.11	-0.46	0.23	0.50	0.76
Amino Acid	C-glycosyltryptophan	-0.07	-0.27	0.14	0.50	0.76
Amino Acid	N2-acetyl,N6,N6-dimethyllysine	0.03	-0.06	0.12	0.52	0.77
Amino Acid	serotonin	-0.10	-0.44	0.24	0.53	0.78
Amino Acid	p-cresol glucuronide*	0.03	-0.07	0.13	0.54	0.78
Amino Acid	N-acetylglutamate	-0.09	-0.40	0.22	0.55	0.79
Amino Acid	tyramine O-sulfate	0.06	-0.16	0.28	0.57	0.80
Amino Acid	S-carboxyethylcysteine	-0.11	-0.53	0.30	0.58	0.81
Amino Acid	lysine	-0.08	-0.36	0.21	0.58	0.81
Amino Acid	5-oxoproline	0.11	-0.32	0.54	0.59	0.81
Amino Acid	hydroxyasparagine**	-0.08	-0.41	0.24	0.59	0.81
Amino Acid	2-methylbutyrylcarnitine (C5)	-0.09	-0.43	0.26	0.59	0.81
Amino Acid	glycine	-0.04	-0.18	0.11	0.60	0.81
Amino Acid	kynurenine	0.06	-0.18	0.30	0.61	0.82
Amino Acid	2-hydroxyphenylacetate	-0.06	-0.31	0.19	0.61	0.82
Amino Acid	cysteinylglycine	-0.07	-0.36	0.22	0.62	0.82
Amino Acid	N-formylmethionine	-0.07	-0.39	0.24	0.62	0.82
Amino Acid	indolebutyrate	0.05	-0.15	0.24	0.63	0.83

Amino Acid	isobutyrylcarnitine (C4)	-0.02	-0.11	0.07	0.63	0.83
Amino Acid	imidazole propionate	-0.05	-0.25	0.16	0.63	0.83
Amino Acid	S-methylcysteine	-0.03	-0.14	0.09	0.64	0.83
Amino Acid	beta-hydroxyisovaleroylcarnitine	-0.05	-0.26	0.16	0.64	0.83
Amino Acid	S-adenosylhomocysteine (SAH)	0.08	-0.30	0.47	0.64	0.83
Amino Acid	dimethylarginine (SDMA + ADMA)	-0.08	-0.45	0.29	0.65	0.84
Amino Acid	3-methyl-2-oxovalerate	-0.07	-0.39	0.25	0.65	0.84
Amino Acid	N-methyltaurine	-0.03	-0.18	0.11	0.65	0.84
Amino Acid	5-hydroxyindole sulfate	0.06	-0.21	0.32	0.66	0.84
Amino Acid	N-acetylmethionine	0.04	-0.17	0.26	0.67	0.85
Amino Acid	dopamine 4-sulfate	-0.06	-0.32	0.21	0.67	0.85
Amino Acid	2-hydroxybutyrate/2-hydroxyisobutyrate	0.03	-0.10	0.16	0.67	0.85
Amino Acid	indole-3-carboxylate	0.06	-0.21	0.32	0.67	0.85
Amino Acid	phenylacetate	0.03	-0.11	0.17	0.68	0.86
Amino Acid	2-hydroxy-4-(methylthio)butanoic acid	-0.02	-0.15	0.10	0.68	0.86
Amino Acid	3-indoxyl sulfate	-0.03	-0.17	0.12	0.69	0.86
Amino Acid	valine	0.10	-0.43	0.63	0.69	0.87
Amino Acid	3-methylglutarylcarnitine (2)	0.02	-0.11	0.15	0.71	0.87
Amino Acid	4-hydroxyphenylpyruvate	-0.14	-0.92	0.64	0.71	0.87
Amino Acid	creatine	-0.03	-0.22	0.16	0.72	0.88
Amino Acid	N-acetyl-1-methylhistidine*	-0.02	-0.15	0.10	0.72	0.88
Amino Acid	anthranilate	0.05	-0.27	0.36	0.76	0.90
Amino Acid	alpha-hydroxyisocaproate	0.05	-0.30	0.39	0.77	0.90
Amino Acid	5-hydroxylysine	0.03	-0.19	0.25	0.78	0.91
Amino Acid	N,N,N-trimethyl-alanylproline betaine (TMAP)	-0.10	-0.88	0.67	0.78	0.91
Amino Acid	N-acetylleucine	-0.05	-0.44	0.34	0.79	0.92
Amino Acid	N-acetylglycine	0.03	-0.19	0.25	0.79	0.92
Amino Acid	glutamate	-0.03	-0.29	0.23	0.80	0.92
Amino Acid	N6,N6,N6-trimethyllysine	-0.02	-0.23	0.18	0.80	0.92
Amino Acid	methionine sulfone	-0.02	-0.21	0.16	0.81	0.92

Amino Acid	2-ketocaprylate	0.04	-0.32	0.41	0.81	0.92
Amino Acid	4-hydroxyphenylacetoylcarnitine	0.01	-0.08	0.10	0.81	0.92
Amino Acid	cysteine sulfinic acid	-0.04	-0.44	0.36	0.82	0.92
Amino Acid	N-acetylvaline	-0.03	-0.30	0.25	0.83	0.93
Amino Acid	3-(4-hydroxyphenyl)lactate	-0.02	-0.18	0.15	0.84	0.93
Amino Acid	glutarylcarnitine (C5-DC)	0.02	-0.17	0.21	0.84	0.93
Amino Acid	acisoga	-0.01	-0.19	0.16	0.86	0.94
Amino Acid	homocitrulline	-0.01	-0.14	0.12	0.87	0.94
Amino Acid	spermidine	-0.04	-0.53	0.46	0.87	0.94
Amino Acid	N-acetyl-aspartyl-glutamate (NAAG)	0.04	-0.51	0.59	0.88	0.95
Amino Acid	pipecolate	0.01	-0.16	0.18	0.89	0.95
Amino Acid	guanidinosuccinate	0.02	-0.24	0.27	0.89	0.95
Amino Acid	5-methylthioadenosine (MTA)	-0.03	-0.43	0.38	0.90	0.96
Amino Acid	trans-4-hydroxyproline	-0.01	-0.15	0.13	0.90	0.96
Amino Acid	picolinate	0.02	-0.33	0.37	0.90	0.96
Amino Acid	homoarginine	0.01	-0.12	0.13	0.91	0.97
Amino Acid	urea	-0.01	-0.15	0.14	0.92	0.97
Amino Acid	7-hydroxyindole sulfate	-0.01	-0.22	0.20	0.92	0.97
Amino Acid	4-acetamidobutanoate	-0.02	-0.52	0.47	0.92	0.97
Amino Acid	pro-hydroxy-pro	-0.02	-0.46	0.42	0.92	0.97
Amino Acid	2-aminoadipate	0.02	-0.48	0.53	0.93	0.97
Amino Acid	glutamine	0.02	-0.40	0.44	0.93	0.97
Amino Acid	betaine	0.00	-0.12	0.11	0.94	0.97
Amino Acid	N,N,N-trimethyl-5-aminovalerate	0.01	-0.21	0.22	0.94	0.98
Amino Acid	indolepropionylglycine	0.00	-0.16	0.15	0.95	0.98
Amino Acid	phenylpyruvate	-0.01	-0.50	0.48	0.96	0.99
Amino Acid	N-acetylputrescine	0.01	-0.33	0.34	0.97	0.99
Amino Acid	cystathionine	-0.01	-0.31	0.30	0.97	0.99
Amino Acid	phenol glucuronide	0.00	-0.05	0.05	0.97	0.99
Amino Acid	phenyllactate (PLA)	0.00	-0.20	0.19	0.98	0.99

Amino Acid	N2,N5-diacetylornithine	0.00	-0.11	0.10	0.98	0.99
Amino Acid	N-acetylasparagine	0.00	-0.15	0.15	0.98	0.99
Amino Acid	N-methylhydroxyproline**	0.00	-0.39	0.39	0.99	1.00
Amino Acid	(N(1) + N(8))-acetylspermidine	0.00	-0.23	0.24	1.00	1.00
Carbohydrate	glucose	-1.28	-1.98	-0.58	0.00	0.04
Carbohydrate	lyxonate	-0.48	-0.80	-0.15	0.01	0.11
Carbohydrate	maltotriose	-0.47	-0.87	-0.06	0.03	0.25
Carbohydrate	xylose	-0.46	-0.93	0.00	0.05	0.35
Carbohydrate	glycerate	-0.19	-0.38	0.00	0.05	0.36
Carbohydrate	mannitol/sorbitol	0.12	-0.01	0.25	0.07	0.39
Carbohydrate	N-acetylneuraminate	-0.16	-0.35	0.02	0.08	0.40
Carbohydrate	glucuronate	-0.23	-0.57	0.10	0.15	0.54
Carbohydrate	erythronate*	-0.23	-0.65	0.19	0.26	0.63
Carbohydrate	lactate	-0.28	-0.82	0.27	0.30	0.65
Carbohydrate	ribitol	-0.27	-0.82	0.28	0.31	0.65
Carbohydrate	maltose	-0.18	-0.60	0.23	0.37	0.70
Carbohydrate	galactonate	-0.29	-0.98	0.39	0.37	0.70
Carbohydrate	arabonate/xylonate	-0.08	-0.26	0.11	0.39	0.70
Carbohydrate	ribonate	-0.20	-0.72	0.32	0.42	0.71
Carbohydrate	N-acetylglucosamine/N-acetylgalactosamine	-0.17	-0.63	0.28	0.42	0.72
Carbohydrate	arabitol/xylitol	-0.13	-0.47	0.22	0.44	0.73
Carbohydrate	1,5-anhydroglucitol (1,5-AG)	-0.07	-0.30	0.15	0.49	0.75
Carbohydrate	pyruvate	-0.27	-1.12	0.59	0.52	0.77
Carbohydrate	fructose	0.19	-0.80	1.18	0.69	0.87
Carbohydrate	sucrose	0.03	-0.19	0.26	0.75	0.89
Carbohydrate	3-phosphoglycerate	0.08	-0.58	0.74	0.80	0.92
Carbohydrate	mannose	-0.04	-0.36	0.28	0.81	0.92
Carbohydrate	N-acetylglucosaminylasparagine	-0.02	-0.40	0.36	0.90	0.96
Cofactors and Vitamins	gamma-CEHC	-0.12	-0.22	-0.01	0.03	0.26

Cofactors and Vitamins	bilirubin (E,E)*	-0.56	-1.09	-0.04	0.04	0.32
Cofactors and Vitamins	pyridoxal	-0.44	-0.85	-0.03	0.04	0.32
Cofactors and Vitamins	pantoate	-0.46	-0.94	0.03	0.06	0.38
Cofactors and Vitamins	beta-cryptoxanthin	-0.18	-0.39	0.02	0.07	0.40
Cofactors and Vitamins	carotene diol (1)	0.10	-0.01	0.21	0.08	0.40
Cofactors and Vitamins	gamma-CEHC glucuronide*	-0.10	-0.23	0.03	0.11	0.49
Cofactors and Vitamins	I-urobilinogen	-0.18	-0.41	0.05	0.11	0.49
Cofactors and Vitamins	gulonate*	-0.23	-0.53	0.07	0.13	0.51
Cofactors and Vitamins	trigonelline (N'-methylnicotinate)	-0.09	-0.21	0.03	0.13	0.51
Cofactors and Vitamins	pantothenate	-0.12	-0.29	0.05	0.15	0.54
Cofactors and Vitamins	carotene diol (3)	0.10	-0.05	0.26	0.18	0.56
Cofactors and Vitamins	alpha-CEHC glucuronide*	-0.11	-0.27	0.05	0.18	0.56
Cofactors and Vitamins	ascorbic acid 2-sulfate	-0.31	-0.81	0.19	0.20	0.57
Cofactors and Vitamins	alpha-CMBHC glucuronide	-0.07	-0.19	0.05	0.21	0.59
Cofactors and Vitamins	alpha-CEHC sulfate	-0.09	-0.23	0.06	0.22	0.60
Cofactors and Vitamins	delta-CEHC	-0.05	-0.13	0.03	0.25	0.61
Cofactors and Vitamins	ascorbic acid 3-sulfate*	-0.09	-0.25	0.07	0.25	0.61
Cofactors and Vitamins	retinol (vitamin A)	-0.11	-0.31	0.09	0.26	0.62
Cofactors and Vitamins	heme	2.27	-2.20	6.74	0.30	0.65
Cofactors and Vitamins	carotene diol (2)	0.08	-0.09	0.24	0.33	0.67
Cofactors and Vitamins	flavin adenine dinucleotide (FAD)	-0.20	-0.69	0.29	0.39	0.70

Cofactors and Vitamins	retinal	-0.24	-0.89	0.41	0.44	0.73
Cofactors and Vitamins	N1-methyl-2-pyridone-5-carboxamide	-0.05	-0.19	0.09	0.45	0.73
Cofactors and Vitamins	threonate	-0.10	-0.38	0.18	0.46	0.74
Cofactors and Vitamins	2-O-methylascorbic acid	-0.04	-0.18	0.09	0.52	0.77
Cofactors and Vitamins	bilirubin (E,Z or Z,E)*	0.09	-0.21	0.40	0.53	0.77
Cofactors and Vitamins	biliverdin	-0.06	-0.29	0.17	0.58	0.81
Cofactors and Vitamins	bilirubin (Z,Z)	-0.06	-0.29	0.17	0.59	0.81
Cofactors and Vitamins	pyridoxate	-0.02	-0.17	0.12	0.73	0.88
Cofactors and Vitamins	1-methylnicotinamide	-0.05	-0.32	0.23	0.73	0.88
Cofactors and Vitamins	quinolinate	0.03	-0.16	0.21	0.76	0.90
Cofactors and Vitamins	gamma-tocopherol/beta-tocopherol	0.02	-0.13	0.17	0.81	0.92
Cofactors and Vitamins	alpha-tocopherol	-0.03	-0.32	0.25	0.81	0.92
Cofactors and Vitamins	oxalate (ethanedioate)	0.00	-0.22	0.21	0.96	0.99
Cofactors and Vitamins	delta-tocopherol	0.00	-0.09	0.09	1.00	1.00
Energy	fumarate	-0.38	-0.66	-0.09	0.01	0.16
Energy	malate	-0.21	-0.37	-0.04	0.02	0.20
Energy	alpha-ketoglutarate	-0.23	-0.65	0.19	0.27	0.63
Energy	succinate	-0.12	-0.39	0.16	0.38	0.70
Energy	citrate	-0.46	-1.55	0.64	0.39	0.70
Energy	citraconate/glutaconate	0.07	-0.14	0.27	0.50	0.76
Energy	2-methylcitrate/homocitrate	-0.13	-0.52	0.27	0.50	0.76
Energy	phosphate	-0.05	-0.21	0.11	0.52	0.77
Energy	aconitate [cis or trans]	-0.03	-0.30	0.23	0.79	0.92
Energy	isocitric lactone	0.03	-0.19	0.24	0.80	0.92

Energy	succinylcarnitine (C4-DC)	0.03	-0.30	0.37	0.83	0.93
Lipid	3-hydroxydecanoate	-0.69	-0.89	-0.48	0.00	0.00
Lipid	tetradecadienoate (14:2)*	-0.66	-0.88	-0.43	0.00	0.00
Lipid	dodecadienoate (12:2)*	-0.63	-0.85	-0.42	0.00	0.00
Lipid	octanoylcarnitine (C8)	-0.40	-0.54	-0.26	0.00	0.00
Lipid	3-hydroxylaurate	-0.60	-0.82	-0.38	0.00	0.00
Lipid	3-hydroxydecanoylcarnitine	-0.68	-0.93	-0.44	0.00	0.00
Lipid	hexadecadienoate (16:2n6)	-1.14	-1.56	-0.71	0.00	0.01
Lipid	16-hydroxypalmitate	-0.53	-0.74	-0.32	0.00	0.01
Lipid	linolenate [alpha or gamma; (18:3n3 or 6)]	-1.06	-1.48	-0.64	0.00	0.01
Lipid	laurate (12:0)	-0.89	-1.24	-0.53	0.00	0.01
Lipid	hexanoylcarnitine (C6)	-0.44	-0.62	-0.26	0.00	0.01
Lipid	(12 or 13)-methylmyristate (a15:0 or i15:0)	-0.85	-1.20	-0.50	0.00	0.01
Lipid	palmitoleate (16:1n7)	-1.17	-1.66	-0.68	0.00	0.01
Lipid	myristate (14:0)	-0.77	-1.09	-0.45	0.00	0.01
Lipid	caprate (10:0)	-0.73	-1.04	-0.42	0.00	0.01
Lipid	myristoleate (14:1n5)	-0.83	-1.19	-0.47	0.00	0.01
Lipid	linoleate (18:2n6)	-1.19	-1.71	-0.67	0.00	0.01
Lipid	pentadecanoate (15:0)	-1.39	-2.00	-0.78	0.00	0.01
Lipid	3-hydroxyoctanoylcarnitine (1)	-0.43	-0.62	-0.24	0.00	0.01
Lipid	10-heptadecenoate (17:1n7)	-1.91	-2.77	-1.05	0.00	0.01
Lipid	oleate/vaccenate (18:1)	-1.27	-1.84	-0.70	0.00	0.01
Lipid	3-hydroxyoctanoate	-0.42	-0.62	-0.23	0.00	0.02
Lipid	laurylcarnitine (C12)	-0.48	-0.69	-0.26	0.00	0.02
Lipid	myristoleoylcarnitine (C14:1)*	-0.50	-0.74	-0.27	0.00	0.02
Lipid	decanoylcarnitine (C10)	-0.38	-0.57	-0.20	0.00	0.02
Lipid	3-hydroxymyristate	-0.30	-0.45	-0.16	0.00	0.02
Lipid	stearidonate (18:4n3)	-0.53	-0.80	-0.27	0.00	0.03
Lipid	2-hydroxydecanoate	-0.56	-0.84	-0.28	0.00	0.03
Lipid	1-margaroylglycerol (17:0)	-0.99	-1.48	-0.49	0.00	0.03

Lipid	palmitate (16:0)	-1.27	-1.92	-0.62	0.00	0.03
Lipid	(2 or 3)-decenoate (10:1n7 or n8)	-0.56	-0.85	-0.27	0.00	0.03
Lipid	margarate (17:0)	-1.00	-1.52	-0.48	0.00	0.03
Lipid	glycolithocholate	-0.46	-0.70	-0.22	0.00	0.03
Lipid	5-dodecenoylcarnitine (C12:1)	-0.77	-1.19	-0.36	0.00	0.04
Lipid	docosapentaenoate (n3 DPA; 22:5n3)	-0.81	-1.25	-0.38	0.00	0.04
Lipid	dihomo-linoleate (20:2n6)	-0.69	-1.07	-0.32	0.00	0.04
Lipid	dodecanedioate (C12-DC)	-0.29	-0.45	-0.13	0.00	0.04
Lipid	cis-4-decenoate (10:1n6)*	-0.65	-1.01	-0.29	0.00	0.04
Lipid	10-nonadecenoate (19:1n9)	-0.49	-0.76	-0.22	0.00	0.04
Lipid	hexanoylglycine	-0.19	-0.30	-0.08	0.00	0.05
Lipid	cis-4-decenoylcarnitine (C10:1)	-0.49	-0.77	-0.21	0.00	0.05
Lipid	12,13-DiHOME	-0.58	-0.91	-0.25	0.00	0.05
Lipid	(14 or 15)-methylpalmitate (a17:0 or i17:0)	-0.46	-0.72	-0.19	0.00	0.05
Lipid	eicosapentaenoate (EPA; 20:5n3)	-0.54	-0.85	-0.23	0.00	0.05
Lipid	estrone 3-sulfate	-0.19	-0.30	-0.08	0.00	0.06
Lipid	taurolithocholate 3-sulfate	-0.17	-0.27	-0.07	0.00	0.06
Lipid	nonanoylcarnitine (C9)	-0.30	-0.48	-0.12	0.00	0.06
Lipid	glycoursodeoxycholate	-0.29	-0.46	-0.11	0.00	0.07
Lipid	tetradecanedioate (C14-DC)	-0.32	-0.51	-0.12	0.00	0.07
Lipid	2-hydroxyoctanoate	-0.26	-0.42	-0.10	0.00	0.07
Lipid	adrenate (22:4n6)	-0.85	-1.37	-0.33	0.00	0.07
Lipid	dihomo-linolenate (20:3n3 or n6)	-0.63	-1.01	-0.24	0.00	0.07
Lipid	butyrylcarnitine (C4)	-0.19	-0.31	-0.07	0.00	0.08
Lipid	docosadienoate (22:2n6)	-0.41	-0.66	-0.15	0.00	0.08
Lipid	taurochenodeoxycholic acid 3-sulfate	-0.11	-0.18	-0.04	0.00	0.09
Lipid	5-dodecenoate (12:1n7)	-0.56	-0.93	-0.20	0.00	0.09
Lipid	docosatrienoate (22:3n3)	-0.30	-0.50	-0.10	0.01	0.10
Lipid	eicosenoate (20:1)	-0.68	-1.13	-0.23	0.01	0.10
Lipid	3-hydroxyoctanoylcarnitine (2)	-0.40	-0.66	-0.13	0.01	0.10

Lipid	taurochenodeoxycholate	-0.59	-0.99	-0.20	0.01	0.11
Lipid	docosahexaenoate (DHA; 22:6n3)	-0.53	-0.89	-0.17	0.01	0.11
Lipid	heneicosapentaenoate (21:5n3)	-0.42	-0.70	-0.14	0.01	0.11
Lipid	hexadecanedioate (C16-DC)	-0.31	-0.52	-0.10	0.01	0.11
Lipid	dodecenedioate (C12:1-DC)*	-0.26	-0.43	-0.08	0.01	0.11
Lipid	3-hydroxydodecanedioate*	-0.32	-0.54	-0.10	0.01	0.12
Lipid	1-linoleoyl-2-linolenoyl-GPC (18:2/18:3)*	-0.53	-0.90	-0.16	0.01	0.12
Lipid	glycolithocholate sulfate*	-0.12	-0.20	-0.03	0.01	0.14
Lipid	3-hydroxybutyrate (BHBA)	-0.43	-0.75	-0.12	0.01	0.15
Lipid	glycocholate	-0.31	-0.53	-0.08	0.01	0.15
Lipid	1,2-dilinoleoyl-GPC (18:2/18:2)	-0.37	-0.64	-0.10	0.01	0.15
Lipid	palmitoleoylcarnitine (C16:1)*	-0.32	-0.56	-0.08	0.01	0.15
Lipid	9-hydroxystearate	-0.33	-0.57	-0.08	0.01	0.15
Lipid	stearate (18:0)	-0.77	-1.35	-0.19	0.01	0.16
Lipid	glycerol	-0.59	-1.04	-0.15	0.01	0.16
Lipid	N-palmitoyltaurine	0.52	0.12	0.92	0.01	0.18
Lipid	hexadecenedioate (C16:1-DC)*	-0.17	-0.31	-0.04	0.01	0.18
Lipid	1-linoleoyl-GPA (18:2)*	-0.45	-0.80	-0.10	0.01	0.18
Lipid	docosatrienoate (22:3n6)*	-0.44	-0.78	-0.10	0.01	0.18
Lipid	arachidonate (20:4n6)	-1.04	-1.84	-0.23	0.01	0.18
Lipid	10-undecenoate (11:1n1)	-0.17	-0.31	-0.04	0.02	0.18
Lipid	butyrate/isobutyrate (4:0)	-0.33	-0.59	-0.07	0.02	0.18
Lipid	tetradecadienedioate (C14:2-DC)*	-0.16	-0.29	-0.03	0.02	0.19
Lipid	glycodeoxycholate	-0.20	-0.35	-0.04	0.02	0.20
Lipid	pristanate	-0.38	-0.68	-0.08	0.02	0.20
Lipid	tauro-beta-muricholate	-0.14	-0.25	-0.03	0.02	0.20
Lipid	N-octanoylglycine	-0.34	-0.62	-0.06	0.02	0.21
Lipid	2-hydroxylaurate	-0.27	-0.50	-0.05	0.02	0.21
Lipid	glycohyocholate	-0.27	-0.49	-0.05	0.02	0.21
Lipid	myristoylcarnitine (C14)	-0.21	-0.39	-0.04	0.02	0.21

Lipid	phosphoethanolamine	-0.45	-0.82	-0.08	0.02	0.21
Lipid	nonadecanoate (19:0)	-0.57	-1.06	-0.09	0.02	0.23
Lipid	linoleoyl ethanolamide	-0.45	-0.84	-0.06	0.03	0.24
Lipid	taurodeoxycholic acid 3-sulfate	-0.20	-0.38	-0.02	0.03	0.26
Lipid	trans-2-hexenoylglycine	-0.23	-0.43	-0.02	0.03	0.28
Lipid	docosapentaenoate (n6 DPA; 22:5n6)	-0.41	-0.77	-0.04	0.03	0.29
Lipid	3beta-hydroxy-5-cholenoate	-0.24	-0.46	-0.02	0.03	0.30
Lipid	isoursodeoxycholate	-0.06	-0.12	0.00	0.04	0.31
Lipid	1-(1-enyl-palmitoyl)-2-linoleoyl-GPC (P-16:0/18:2)*	-0.13	-0.26	-0.01	0.04	0.31
Lipid	5-hydroxyhexanoate	-0.40	-0.77	-0.02	0.04	0.32
Lipid	5alpha-androstan-3alpha,17beta-diol monosulfate (1)	-0.06	-0.12	0.00	0.04	0.32
Lipid	1-stearoyl-2-oleoyl-GPE (18:0/18:1)	-0.23	-0.44	-0.01	0.04	0.32
Lipid	N-linoleoylglycine	-0.44	-0.87	-0.02	0.04	0.32
Lipid	1-oleoyl-2-linoleoyl-GPE (18:1/18:2)*	-0.21	-0.42	-0.01	0.04	0.33
Lipid	7alpha-hydroxy-3-oxo-4-cholestenoate (7-Hoca)	-0.24	-0.47	-0.01	0.04	0.33
Lipid	5alpha-pregnan-3beta,20alpha-diol monosulfate (2)	-0.10	-0.19	0.00	0.04	0.33
Lipid	sphingomyelin (d18:2/14:0, d18:1/14:1)*	-0.20	-0.39	0.00	0.05	0.33
Lipid	3-hydroxysebacate	-0.15	-0.31	0.00	0.05	0.34
Lipid	myo-inositol	-0.39	-0.77	0.00	0.05	0.35
Lipid	sphingomyelin (d17:1/14:0, d16:1/15:0)*	-0.16	-0.32	0.00	0.05	0.35
Lipid	glycochenodeoxycholate	-0.23	-0.45	0.00	0.05	0.35
Lipid	N-oleoyltaurine	-0.25	-0.50	0.00	0.05	0.35
Lipid	5alpha-pregnan-3beta,20beta-diol monosulfate (1)	-0.11	-0.22	0.00	0.05	0.36
Lipid	1-stearoyl-2-dihomo-linolenoyl-GPE (18:0/20:3n3 or 6)*	-0.14	-0.28	0.00	0.06	0.36
Lipid	21-hydroxypregnenolone monosulfate (1)	0.26	-0.01	0.52	0.06	0.37
Lipid	9,10-DiHOME	-0.18	-0.36	0.01	0.06	0.37
Lipid	lithocholate sulfate (1)	-0.14	-0.29	0.01	0.06	0.38
Lipid	glycodeoxycholate 3-sulfate	-0.10	-0.20	0.00	0.06	0.38
Lipid	sphingomyelin (d18:1/20:0, d16:1/22:0)*	-0.37	-0.75	0.02	0.06	0.38
Lipid	glyco-beta-muricholate**	-0.13	-0.27	0.01	0.06	0.38

Lipid	octadecenedioate (C18:1-DC)	-0.11	-0.22	0.01	0.06	0.38
Lipid	taurodeoxycholate	-0.28	-0.58	0.02	0.06	0.38
Lipid	sebacate (C10-DC)	-0.20	-0.40	0.01	0.06	0.39
Lipid	ursodeoxycholate	-0.18	-0.37	0.01	0.06	0.39
Lipid	alpha-hydroxycaproate	0.51	-0.04	1.06	0.07	0.39
Lipid	1-palmitoyl-2-palmitoleoyl-GPE (16:0/16:1)*	-0.18	-0.37	0.01	0.07	0.39
Lipid	3beta-hydroxy-5-cholestenoate	-0.34	-0.71	0.03	0.07	0.40
Lipid	glutarate (C5-DC)	-0.11	-0.24	0.01	0.07	0.40
Lipid	choline phosphate	-0.33	-0.70	0.04	0.08	0.40
Lipid	taurocholate	-0.15	-0.33	0.02	0.08	0.40
Lipid	glycocholate glucuronide (1)	-0.13	-0.28	0.02	0.08	0.41
Lipid	octadecenedioylcarnitine (C18:1-DC)*	-0.09	-0.18	0.01	0.08	0.41
Lipid	1-myristoyl-2-palmitoyl-GPC (14:0/16:0)	-0.14	-0.31	0.02	0.09	0.43
Lipid	1-margaroyl-2-arachidonoyl-GPC (17:0/20:4)*	-0.17	-0.38	0.03	0.09	0.43
Lipid	linoleoylcarnitine (C18:2)*	-0.24	-0.51	0.04	0.09	0.44
Lipid	glycosyl-N-behenoyl-sphingadienine (d18:2/22:0)*	-0.23	-0.51	0.05	0.10	0.46
Lipid	glycochenodeoxycholate 3-sulfate	-0.07	-0.15	0.01	0.10	0.46
Lipid	glycosyl-N-palmitoyl-sphingosine (d18:1/16:0)	-0.21	-0.45	0.04	0.10	0.46
Lipid	propionylcarnitine (C3)	-0.13	-0.29	0.03	0.10	0.47
Lipid	eicosenedioate (C20:1-DC)*	-0.16	-0.35	0.04	0.10	0.47
Lipid	androstenediol (3beta,17beta) monosulfate (1)	-0.04	-0.09	0.01	0.10	0.47
Lipid	tauroursodeoxycholate	-0.24	-0.55	0.06	0.10	0.47
Lipid	1-(1-enyl-palmitoyl)-2-oleoyl-GPC (P-16:0/18:1)*	-0.14	-0.31	0.03	0.11	0.48
Lipid	heptanoate (7:0)	-0.37	-0.82	0.09	0.11	0.48
Lipid	1-stearoyl-2-dihomo-linolenoyl-GPC (18:0/20:3n3 or 6)*	-0.10	-0.23	0.03	0.11	0.49
Lipid	palmitoyl dihydrosphingomyelin (d18:0/16:0)*	-0.20	-0.46	0.05	0.11	0.49
Lipid	1-palmityl-GPE (O-16:0)*	0.22	-0.06	0.51	0.11	0.49
Lipid	1-(1-enyl-palmitoyl)-2-docosahexaenoyl-GPC (P-16:0/22:6)*	-0.11	-0.26	0.03	0.12	0.49
Lipid	androstenediol (3alpha, 17alpha) monosulfate (3)	-0.03	-0.07	0.01	0.12	0.51
Lipid	1-pentadecanoyl-2-linoleoyl-GPC (15:0/18:2)*	-0.15	-0.35	0.05	0.12	0.51

Lipid	sphingomyelin (d18:2/16:0, d18:1/16:1)*	-0.24	-0.55	0.07	0.12	0.51
Lipid	3-hydroxyadipate	-0.33	-0.76	0.10	0.12	0.51
Lipid	2-stearoyl-GPE (18:0)*	-0.35	-0.81	0.11	0.12	0.51
Lipid	N-acetyl-2-aminooctanoate*	0.13	-0.04	0.30	0.13	0.51
Lipid	pregnenediol sulfate (C21H34O5S)*	-0.08	-0.19	0.03	0.13	0.51
Lipid	suberoylcarnitine (C8-DC)	0.12	-0.04	0.29	0.13	0.52
Lipid	deoxycholic acid 12-sulfate*	-0.11	-0.26	0.04	0.13	0.52
Lipid	N-palmitoylglycine	-0.27	-0.63	0.09	0.13	0.52
Lipid	arachidate (20:0)	-0.27	-0.64	0.09	0.13	0.52
Lipid	1-palmitoyl-2-eicosapentaenoyl-GPE (16:0/20:5)*	-0.12	-0.29	0.04	0.13	0.52
Lipid	ceramide (d18:1/14:0, d16:1/16:0)*	-0.16	-0.37	0.06	0.14	0.52
Lipid	1-palmitoyl-2-linoleoyl-GPE (16:0/18:2)	-0.14	-0.33	0.05	0.14	0.52
Lipid	octadecanedioate (C18-DC)	-0.17	-0.41	0.06	0.14	0.52
Lipid	glycosyl-N-stearoyl-sphingosine (d18:1/18:0)	-0.24	-0.57	0.09	0.14	0.52
Lipid	cholate	-0.30	-0.72	0.12	0.15	0.54
Lipid	1-myristoyl-2-arachidonoyl-GPC (14:0/20:4)*	-0.07	-0.18	0.03	0.15	0.54
Lipid	dihomo-linolenoylcarnitine (C20:3n3 or 6)*	-0.22	-0.54	0.09	0.16	0.54
Lipid	1-eicosenoyl-GPE (20:1)*	-0.36	-0.88	0.16	0.16	0.54
Lipid	1-stearoyl-2-linoleoyl-GPE (18:0/18:2)*	-0.11	-0.26	0.05	0.16	0.54
Lipid	linoleoyl-linolenoyl-glycerol (18:2/18:3) [2]*	-0.22	-0.54	0.10	0.16	0.54
Lipid	1,2-dilinoleoyl-GPE (18:2/18:2)*	-0.29	-0.71	0.13	0.16	0.55
Lipid	butyrylglycine	-0.09	-0.21	0.04	0.16	0.55
Lipid	1-lignoceroyl-GPC (24:0)	-0.18	-0.44	0.08	0.17	0.55
Lipid	1-palmitoyl-2-palmitoleoyl-GPC (16:0/16:1)*	-0.09	-0.23	0.04	0.17	0.55
Lipid	sphingomyelin (d17:1/16:0, d18:1/15:0, d16:1/17:0)*	-0.19	-0.46	0.09	0.17	0.55
Lipid	1-palmitoyl-2-arachidonoyl-GPC (16:0/20:4n6)	-0.12	-0.30	0.06	0.17	0.56
Lipid	1-linoleoyl-GPG (18:2)*	-0.31	-0.77	0.15	0.17	0.56
Lipid	1-(1-enyl-palmitoyl)-2-linoleoyl-GPE (P-16:0/18:2)*	-0.11	-0.28	0.05	0.17	0.56
Lipid	oleoylcarnitine (C18:1)	-0.20	-0.49	0.10	0.17	0.56
Lipid	azelate (C9-DC)	0.48	-0.23	1.18	0.17	0.56

Lipid	13-HODE + 9-HODE	-0.29	-0.72	0.14	0.17	0.56
Lipid	caproate (6:0)	-0.20	-0.49	0.10	0.17	0.56
Lipid	carnitine	-0.36	-0.89	0.18	0.17	0.56
Lipid	1-(1-enyl-stearoyl)-2-linoleoyl-GPE (P-18:0/18:2)*	-0.11	-0.27	0.05	0.17	0.56
Lipid	5alpha-androstan-3beta,17beta-diol monosulfate (2)	-0.05	-0.13	0.03	0.18	0.56
Lipid	1-margaroyl-GPE (17:0)*	-0.30	-0.75	0.15	0.18	0.56
Lipid	cis-3,4-methyleneheptanoylglycine	-0.11	-0.29	0.06	0.18	0.56
Lipid	(R)-3-hydroxybutyrylcarnitine	0.39	-0.21	0.99	0.19	0.56
Lipid	3beta,7alpha-dihydroxy-5-cholestenoate	-0.32	-0.82	0.17	0.19	0.56
Lipid	1-arachidoyl-GPC (20:0)	0.07	-0.04	0.18	0.19	0.56
Lipid	sphingomyelin (d18:1/22:2, d18:2/22:1, d16:1/24:2)*	-0.13	-0.33	0.07	0.19	0.56
Lipid	3-hydroxybutyroylglycine**	0.12	-0.07	0.30	0.20	0.56
Lipid	5alpha-androstan-3alpha,17alpha-diol monosulfate	-0.10	-0.26	0.06	0.20	0.56
Lipid	tridecenedioate (C13:1-DC)*	-0.11	-0.29	0.07	0.20	0.57
Lipid	dihomo-linolenoyl-choline	0.49	-0.29	1.27	0.20	0.57
Lipid	arachidoylcarnitine (C20)*	-0.32	-0.83	0.20	0.21	0.58
Lipid	2-arachidonoylglycerol (20:4)	0.26	-0.16	0.67	0.21	0.58
Lipid	corticosterone	0.39	-0.24	1.02	0.21	0.58
Lipid	1-stearoyl-2-arachidonoyl-GPS (18:0/20:4)	-0.34	-0.90	0.22	0.21	0.58
Lipid	1-margaroyl-2-linoleoyl-GPC (17:0/18:2)*	-0.19	-0.49	0.12	0.22	0.59
Lipid	1-(1-enyl-palmitoyl)-2-arachidonoyl-GPC (P-16:0/20:4)*	-0.09	-0.24	0.06	0.22	0.59
Lipid	1-palmitoyl-2-pentadecanoyl-GPC (16:0/15:0)*	-0.20	-0.53	0.13	0.22	0.59
Lipid	glycosyl ceramide (d18:1/20:0, d16:1/22:0)*	-0.20	-0.52	0.13	0.22	0.59
Lipid	stearoyl sphingomyelin (d18:1/18:0)	-0.16	-0.44	0.11	0.22	0.60
Lipid	1-stearoyl-2-linoleoyl-GPC (18:0/18:2)*	-0.17	-0.45	0.11	0.22	0.60
Lipid	glycocholate sulfate	-0.02	-0.07	0.02	0.23	0.60
Lipid	N-palmitoyl-sphingadienine (d18:2/16:0)*	-0.25	-0.68	0.18	0.23	0.60
Lipid	1-(1-enyl-stearoyl)-2-docosahexaenoyl-GPE (P-18:0/22:6)*	-0.11	-0.31	0.08	0.23	0.60
Lipid	linoleoyl-linoleoyl-glycerol (18:2/18:2) [2]*	0.19	-0.13	0.50	0.23	0.60
Lipid	1-(1-enyl-stearoyl)-GPE (P-18:0)*	0.23	-0.16	0.63	0.23	0.60

Lipid	oleoyl ethanolamide	-0.43	-1.15	0.30	0.23	0.60
Lipid	propionylglycine	-0.34	-0.91	0.24	0.23	0.60
Lipid	cortisol	0.24	-0.18	0.66	0.24	0.60
Lipid	1-pentadecanoyl-2-arachidonoyl-GPC (15:0/20:4)*	-0.08	-0.21	0.06	0.24	0.60
Lipid	hydroxy-CMPF*	-0.04	-0.11	0.03	0.24	0.60
Lipid	cerotoylcarnitine (C26)*	-0.13	-0.36	0.10	0.24	0.60
Lipid	cholic acid glucuronide	-0.05	-0.13	0.03	0.24	0.60
Lipid	1-palmitoyl-2-(hydroxylinoleoyl)-GPC (16:0/18:2(OH))*	-0.35	-0.98	0.27	0.25	0.61
Lipid	(S)-3-hydroxybutyrylcarnitine	-0.09	-0.26	0.07	0.25	0.61
Lipid	linoleoyl-arachidonoyl-glycerol (18:2/20:4) [1]*	-0.16	-0.44	0.12	0.25	0.62
Lipid	1-(1-enyl-palmitoyl)-2-palmitoyl-GPC (P-16:0/16:0)*	-0.14	-0.39	0.11	0.26	0.62
Lipid	androstenediol (3beta,17beta) disulfate (2)	-0.06	-0.17	0.05	0.26	0.63
Lipid	taurocholenate sulfate*	-0.07	-0.19	0.06	0.26	0.63
Lipid	undecenoylcarnitine (C11:1)	-0.14	-0.39	0.12	0.27	0.63
Lipid	palmitoyl sphingomyelin (d18:1/16:0)	-0.25	-0.71	0.21	0.27	0.63
Lipid	1-arachidoyl-2-arachidonoyl-GPC (20:0/20:4)*	-0.24	-0.69	0.21	0.27	0.63
Lipid	methylmalonate (MMA)	-0.22	-0.63	0.19	0.27	0.63
Lipid	oleoylcholine	0.35	-0.30	1.00	0.27	0.63
Lipid	deoxycholate	-0.09	-0.26	0.08	0.28	0.63
Lipid	caprylate (8:0)	-0.23	-0.68	0.21	0.28	0.64
Lipid	docosadioate (C22-DC)	0.28	-0.25	0.81	0.28	0.64
Lipid	1-stearoyl-GPG (18:0)	-0.28	-0.83	0.26	0.28	0.64
Lipid	N-palmitoyl-sphingosine (d18:1/16:0)	-0.18	-0.53	0.17	0.28	0.64
Lipid	androsterone sulfate	-0.07	-0.19	0.06	0.28	0.64
Lipid	1-(1-enyl-palmitoyl)-2-docosahexaenoyl-GPE (P-16:0/22:6)*	-0.09	-0.27	0.09	0.29	0.64
Lipid	sphingomyelin (d18:1/22:1, d18:2/22:0, d16:1/24:1)*	-0.19	-0.57	0.18	0.29	0.64
Lipid	arachidonoylcarnitine (C20:4)	-0.15	-0.43	0.14	0.29	0.65
Lipid	3-carboxy-4-methyl-5-pentyl-2-furanpropionate (3-CMPFP)**	-0.08	-0.23	0.08	0.30	0.65
Lipid	sphingomyelin (d18:1/25:0, d19:0/24:1, d20:1/23:0, d19:1/24:0)*	-0.07	-0.22	0.07	0.30	0.65

Lipid	sphingadienine	0.20	-0.20	0.60	0.31	0.65
Lipid	1-palmitoyl-2-linoleoyl-GPI (16:0/18:2)	0.24	-0.25	0.73	0.31	0.65
Lipid	N-stearoyl-sphingosine (d18:1/18:0)*	-0.11	-0.34	0.12	0.31	0.66
Lipid	21-hydroxypregnenolone disulfate	0.06	-0.06	0.18	0.32	0.66
Lipid	1-palmitoyl-2-oleoyl-GPE (16:0/18:1)	-0.12	-0.36	0.13	0.32	0.67
Lipid	3-hydroxyoleate*	-0.34	-1.05	0.37	0.32	0.67
Lipid	1-stearyl-GPE (O-18:0)*	-0.09	-0.28	0.10	0.32	0.67
Lipid	trimethylamine N-oxide	-0.04	-0.11	0.04	0.33	0.67
Lipid	acetylcarnitine (C2)	-0.14	-0.43	0.15	0.33	0.67
Lipid	docosahexaenoylcholine	0.23	-0.27	0.74	0.34	0.68
Lipid	1-docosahexaenoyl-GPE (22:6)*	-0.13	-0.41	0.15	0.34	0.68
Lipid	(16 or 17)-methylstearate (a19:0 or i19:0)	-0.06	-0.19	0.07	0.34	0.68
Lipid	1-dihomo-linolenoyl-GPC (20:3n3 or 6)*	0.15	-0.18	0.47	0.34	0.68
Lipid	1-palmityl-GPC (O-16:0)	0.11	-0.13	0.34	0.35	0.68
Lipid	1-stearoyl-2-docosapentaenoyl-GPC (18:0/22:5n3)*	-0.07	-0.21	0.08	0.35	0.68
Lipid	1-arachidonoyl-GPC (20:4n6)*	0.10	-0.12	0.32	0.35	0.68
Lipid	myristoyl dihydrosphingomyelin (d18:0/14:0)*	-0.08	-0.27	0.10	0.35	0.68
Lipid	oleoyl-linoleoyl-glycerol (18:1/18:2) [1]	-0.22	-0.69	0.26	0.35	0.68
Lipid	1-stearoyl-2-docosapentaenoyl-GPC (18:0/22:5n6)*	-0.08	-0.26	0.10	0.35	0.68
Lipid	sphingomyelin (d18:1/18:1, d18:2/18:0)	-0.12	-0.38	0.15	0.35	0.69
Lipid	lactosyl-N-palmitoyl-sphingosine (d18:1/16:0)	-0.11	-0.35	0.13	0.36	0.69
Lipid	1-palmitoleoylglycerol (16:1)*	0.21	-0.26	0.67	0.36	0.69
Lipid	glycerophosphorylcholine (GPC)	0.24	-0.30	0.77	0.36	0.69
Lipid	maleate	-0.20	-0.67	0.26	0.36	0.69
Lipid	linoleoyl-linoleoyl-glycerol (18:2/18:2) [1]*	-0.10	-0.34	0.13	0.36	0.70
Lipid	1-stearoyl-GPI (18:0)	0.31	-0.40	1.03	0.37	0.70
Lipid	epiandrosterone sulfate	-0.02	-0.07	0.03	0.37	0.70
Lipid	1-oleoyl-GPE (18:1)	-0.12	-0.38	0.15	0.37	0.70
Lipid	1-linoleoyl-2-arachidonoyl-GPE (18:2/20:4)*	0.15	-0.20	0.51	0.37	0.70
Lipid	1-palmityl-2-linoleoyl-GPC (O-16:0/18:2)*	-0.07	-0.25	0.10	0.37	0.70

Lipid	1-palmitoyl-2-docosahexaenoyl-GPE (16:0/22:6)*	-0.07	-0.25	0.10	0.37	0.70
Lipid	5alpha-pregnan-diol disulfate	0.05	-0.07	0.17	0.37	0.70
Lipid	1-palmitoyl-2-linoleoyl-GPC (16:0/18:2)	-0.14	-0.48	0.19	0.37	0.70
Lipid	palmitoylcarnitine (C16)	-0.13	-0.43	0.17	0.37	0.70
Lipid	1-(1-enyl-oleoyl)-GPE (P-18:1)*	-0.21	-0.71	0.28	0.37	0.70
Lipid	glycosyl ceramide (d18:2/24:1, d18:1/24:2)*	-0.10	-0.32	0.13	0.37	0.70
Lipid	1-palmitoleoyl-GPI (16:1)*	0.09	-0.12	0.31	0.38	0.70
Lipid	stearoylcholine*	0.29	-0.40	0.98	0.38	0.70
Lipid	picolinoylglycine	-0.09	-0.29	0.12	0.38	0.70
Lipid	sphingomyelin (d18:2/23:0, d18:1/23:1, d17:1/24:1)*	-0.12	-0.40	0.16	0.38	0.70
Lipid	N-palmitoyl-sphinganine (d18:0/16:0)	-0.11	-0.38	0.16	0.39	0.70
Lipid	pregnanolone/allopregnanolone sulfate	0.14	-0.20	0.49	0.39	0.70
Lipid	1-oleoyl-2-docosahexaenoyl-GPC (18:1/22:6)*	-0.07	-0.25	0.10	0.39	0.70
Lipid	1-palmitoyl-2-eicosapentaenoyl-GPC (16:0/20:5)*	-0.07	-0.24	0.10	0.39	0.70
Lipid	dihomo-linoleoylcarnitine (C20:2)*	-0.13	-0.45	0.19	0.39	0.70
Lipid	1-palmitoyl-2-docosahexaenoyl-GPC (16:0/22:6)	-0.07	-0.23	0.09	0.39	0.70
Lipid	1-stearoyl-GPS (18:0)*	0.21	-0.30	0.71	0.39	0.70
Lipid	1-(1-enyl-stearoyl)-2-oleoyl-GPE (P-18:0/18:1)	-0.09	-0.31	0.13	0.39	0.70
Lipid	1-stearoyl-GPE (18:0)	0.10	-0.14	0.34	0.39	0.70
Lipid	palmitoyl-sphingosine-phosphoethanolamine (d18:1/16:0)	-0.10	-0.35	0.15	0.39	0.70
Lipid	1-pentadecanoyl-2-docosahexaenoyl-GPC (15:0/22:6)*	-0.08	-0.29	0.12	0.40	0.70
Lipid	1-palmityl-2-palmitoyl-GPC (O-16:0/16:0)*	-0.17	-0.58	0.25	0.40	0.70
Lipid	sphinganine	0.38	-0.56	1.32	0.40	0.70
Lipid	chiro-inositol	0.48	-0.70	1.67	0.40	0.70
Lipid	sphingomyelin (d18:1/17:0, d17:1/18:0, d19:1/16:0)	-0.14	-0.49	0.21	0.40	0.70
Lipid	1-(1-enyl-stearoyl)-2-arachidonoyl-GPE (P-18:0/20:4)*	-0.08	-0.27	0.11	0.40	0.71
Lipid	sphingomyelin (d18:2/21:0, d16:2/23:0)*	-0.08	-0.29	0.12	0.40	0.71
Lipid	deoxycholic acid glucuronide	-0.04	-0.13	0.05	0.41	0.71
Lipid	1-palmitoylglycerol (16:0)	0.37	-0.56	1.30	0.41	0.71
Lipid	1-stearoyl-2-docosahexaenoyl-GPE (18:0/22:6)*	0.13	-0.20	0.46	0.41	0.71

Lipid	sphinganine-1-phosphate	0.19	-0.29	0.67	0.42	0.71
Lipid	1-oleoylglycerol (18:1)	0.30	-0.47	1.08	0.42	0.71
Lipid	1-stearoyl-2-oleoyl-GPC (18:0/18:1)	-0.10	-0.38	0.17	0.42	0.72
Lipid	glycocholenate sulfate*	-0.05	-0.17	0.07	0.42	0.72
Lipid	sphingomyelin (d18:1/21:0, d17:1/22:0, d16:1/23:0)*	-0.08	-0.28	0.12	0.43	0.73
Lipid	1-oleoyl-2-arachidonoyl-GPE (18:1/20:4)*	-0.08	-0.29	0.13	0.43	0.73
Lipid	oleoyl-arachidonoyl-glycerol (18:1/20:4) [1]*	0.21	-0.35	0.76	0.44	0.73
Lipid	eicosanedioate (C20-DC)	-0.08	-0.29	0.13	0.44	0.73
Lipid	linoleoylcholine*	0.24	-0.40	0.87	0.44	0.73
Lipid	heptenedioate (C7:1-DC)*	-0.08	-0.28	0.13	0.44	0.73
Lipid	sphingomyelin (d18:0/18:0, d19:0/17:0)*	-0.06	-0.22	0.10	0.44	0.73
Lipid	glycerol 3-phosphate	-0.15	-0.57	0.26	0.45	0.73
Lipid	1-stearoyl-2-linoleoyl-GPI (18:0/18:2)	-0.11	-0.41	0.19	0.45	0.73
Lipid	1-palmitoleoyl-GPC (16:1)*	0.07	-0.13	0.27	0.45	0.73
Lipid	1-stearoyl-2-docosahexaenoyl-GPC (18:0/22:6)	-0.08	-0.30	0.14	0.45	0.73
Lipid	mead acid (20:3n9)	-0.12	-0.46	0.22	0.45	0.73
Lipid	1-palmitoyl-2-dihomo-linolenoyl-GPC (16:0/20:3n3 or 6)*	-0.07	-0.26	0.12	0.46	0.73
Lipid	1-(1-enyl-palmitoyl)-GPC (P-16:0)*	0.10	-0.17	0.36	0.46	0.74
Lipid	sphingomyelin (d18:1/20:1, d18:2/20:0)*	-0.08	-0.29	0.14	0.46	0.74
Lipid	pimeloylcarnitine/3-methyladipoylcarnitine (C7-DC)	-0.05	-0.18	0.09	0.46	0.74
Lipid	1-arachidonylglycerol (20:4)	0.20	-0.36	0.76	0.46	0.74
Lipid	1-dihomo-linoleoyl-GPC (20:2)*	0.14	-0.26	0.55	0.46	0.74
Lipid	sphingosine	-0.27	-1.04	0.50	0.46	0.74
Lipid	1-stearoyl-2-arachidonoyl-GPE (18:0/20:4)	-0.07	-0.26	0.12	0.47	0.74
Lipid	androstenediol (3beta,17beta) disulfate (1)	-0.02	-0.07	0.03	0.47	0.74
Lipid	stearoylcarnitine (C18)	-0.11	-0.43	0.21	0.47	0.74
Lipid	androsterone glucuronide	-0.06	-0.22	0.10	0.47	0.74
Lipid	lactosyl-N-nervonoyl-sphingosine (d18:1/24:1)*	-0.18	-0.70	0.34	0.48	0.75
Lipid	palmitoyl-linoleoyl-glycerol (16:0/18:2) [2]*	0.08	-0.16	0.33	0.48	0.75
Lipid	1-palmityl-2-stearoyl-GPC (O-16:0/18:0)*	-0.15	-0.60	0.30	0.48	0.75

Lipid	3,4-dihydroxybutyrate	-0.16	-0.62	0.31	0.49	0.75
Lipid	1-stearoyl-2-dihomo-linolenoyl-GPI (18:0/20:3n3 or 6)*	-0.09	-0.38	0.19	0.49	0.75
Lipid	1-palmitoyl-GPG (16:0)*	-0.15	-0.62	0.31	0.49	0.75
Lipid	2-hydroxybehenate	0.19	-0.39	0.77	0.50	0.76
Lipid	1-arachidonoyl-GPE (20:4n6)*	-0.06	-0.25	0.13	0.50	0.76
Lipid	dehydroepiandrosterone sulfate (DHEA-S)	-0.01	-0.04	0.02	0.50	0.76
Lipid	1-pentadecanoyl-GPC (15:0)*	0.10	-0.21	0.42	0.50	0.76
Lipid	1-nonadecanoyl-GPC (19:0)	0.07	-0.15	0.28	0.50	0.76
Lipid	sphingosine 1-phosphate	0.11	-0.22	0.43	0.50	0.76
Lipid	17alpha-hydroxypregnanolone glucuronide	-0.05	-0.20	0.10	0.51	0.77
Lipid	1-(1-enyl-palmitoyl)-2-oleoyl-GPE (P-16:0/18:1)*	-0.08	-0.32	0.17	0.51	0.77
Lipid	sphingomyelin (d18:2/24:1, d18:1/24:2)*	-0.09	-0.36	0.19	0.51	0.77
Lipid	1-arachidonoyl-GPI (20:4)*	-0.26	-1.11	0.58	0.52	0.77
Lipid	cis-3,4-methyleneheptanoate	-0.04	-0.18	0.09	0.52	0.77
Lipid	glycoursodeoxycholic acid sulfate (1)	-0.02	-0.07	0.04	0.52	0.77
Lipid	2-arachidonoyl-GPI (20:4)*	0.18	-0.39	0.74	0.52	0.77
Lipid	1-(1-enyl-palmitoyl)-2-arachidonoyl-GPE (P-16:0/20:4)*	-0.06	-0.25	0.13	0.52	0.77
Lipid	1-(1-enyl-palmitoyl)-GPE (P-16:0)*	0.16	-0.36	0.68	0.52	0.77
Lipid	sphingomyelin (d18:1/14:0, d16:1/16:0)*	-0.08	-0.33	0.17	0.52	0.77
Lipid	sphingomyelin (d18:1/19:0, d19:1/18:0)*	-0.06	-0.27	0.14	0.53	0.77
Lipid	2-aminoheptanoate	-0.04	-0.17	0.09	0.53	0.78
Lipid	cholesterol sulfate	-0.06	-0.27	0.15	0.53	0.78
Lipid	3-hydroxy-3-methylglutarate	-0.18	-0.79	0.43	0.54	0.78
Lipid	suberate (C8-DC)	0.08	-0.19	0.34	0.54	0.78
Lipid	sphingomyelin (d18:2/23:1)*	-0.05	-0.24	0.14	0.55	0.79
Lipid	lignoceroylcarnitine (C24)*	-0.06	-0.28	0.16	0.56	0.79
Lipid	1-stearoyl-2-oleoyl-GPS (18:0/18:1)	0.25	-0.64	1.13	0.56	0.79
Lipid	2-linoleoylglycerol (18:2)	0.18	-0.46	0.81	0.56	0.79
Lipid	pregnenetriol disulfate*	-0.02	-0.09	0.05	0.56	0.80
Lipid	sphingomyelin (d18:1/20:2, d18:2/20:1, d16:1/22:2)*	-0.07	-0.32	0.18	0.56	0.80

Lipid	cholesterol	-0.09	-0.43	0.24	0.56	0.80
Lipid	1-stearoyl-2-arachidonoyl-GPI (18:0/20:4)	-0.07	-0.34	0.19	0.57	0.80
Lipid	adipate (C6-DC)	0.07	-0.18	0.31	0.57	0.80
Lipid	1-(1-enyl-stearoyl)-2-docosahexaenoyl-GPC (P-18:0/22:6)*	-0.03	-0.15	0.09	0.57	0.80
Lipid	deoxycarnitine	-0.06	-0.30	0.17	0.57	0.80
Lipid	1-palmitoyl-2-arachidonoyl-GPE (16:0/20:4)*	-0.05	-0.22	0.13	0.58	0.81
Lipid	1,2-dipalmitoyl-GPC (16:0/16:0)	-0.09	-0.44	0.25	0.58	0.81
Lipid	3-hydroxyhexanoylcarnitine (1)	-0.03	-0.17	0.10	0.58	0.81
Lipid	octadecanedioylcarnitine (C18-DC)*	0.05	-0.14	0.24	0.58	0.81
Lipid	tetrahydrocortisone glucuronide (5)	-0.05	-0.22	0.13	0.58	0.81
Lipid	etiocholanolone glucuronide	0.04	-0.10	0.18	0.58	0.81
Lipid	1-palmitoyl-2-oleoyl-GPC (16:0/18:1)	-0.07	-0.33	0.19	0.59	0.81
Lipid	hyocholate	-0.06	-0.30	0.18	0.59	0.81
Lipid	choline	-0.05	-0.26	0.15	0.59	0.81
Lipid	cortisone	0.06	-0.16	0.28	0.59	0.81
Lipid	sphingomyelin (d17:2/16:0, d18:2/15:0)*	-0.06	-0.27	0.16	0.59	0.81
Lipid	1-adrenoyl-GPC (22:4)*	-0.10	-0.50	0.30	0.59	0.81
Lipid	sphingomyelin (d18:1/24:1, d18:2/24:0)*	-0.08	-0.38	0.22	0.60	0.81
Lipid	2-butenoylglycine	-0.10	-0.51	0.31	0.60	0.81
Lipid	1-stearoyl-2-(hydroxylinoleoyl)-GPC (18:0/18:2(OH))*	-0.17	-0.86	0.52	0.60	0.81
Lipid	1-eicosenoyl-GPC (20:1)*	0.08	-0.24	0.39	0.60	0.81
Lipid	chenodeoxycholate	0.08	-0.25	0.41	0.61	0.82
Lipid	arachidonoylcholine	0.17	-0.52	0.85	0.61	0.82
Lipid	tetrahydrocortisol glucuronide	-0.05	-0.26	0.16	0.62	0.82
Lipid	2R,3R-dihydroxybutyrate	-0.08	-0.41	0.25	0.62	0.82
Lipid	2-stearoyl-GPI (18:0)*	0.20	-0.69	1.10	0.64	0.83
Lipid	tricosanoyl sphingomyelin (d18:1/23:0)*	-0.05	-0.28	0.18	0.64	0.83
Lipid	ximenoylcarnitine (C26:1)*	-0.07	-0.37	0.23	0.64	0.83
Lipid	11-ketoetiocholanolone glucuronide	0.03	-0.11	0.17	0.64	0.83
Lipid	pregnanediol-3-glucuronide	-0.03	-0.15	0.09	0.64	0.83

Lipid	1-linoleoyl-GPE (18:2)*	-0.05	-0.29	0.19	0.65	0.84
Lipid	3-hydroxystearate	-0.07	-0.39	0.25	0.65	0.84
Lipid	palmitoyl-arachidonoyl-glycerol (16:0/20:4) [1]*	0.10	-0.37	0.57	0.66	0.85
Lipid	5alpha-androstan-3alpha,17beta-diol monosulfate (2)	-0.01	-0.08	0.05	0.67	0.85
Lipid	1-stearoyl-GPC (18:0)	0.04	-0.18	0.27	0.68	0.86
Lipid	oleoyl-oleoyl-glycerol (18:1/18:1) [2]*	0.12	-0.49	0.73	0.68	0.86
Lipid	1-linoleoyl-2-arachidonoyl-GPC (18:2/20:4n6)*	0.07	-0.30	0.45	0.68	0.86
Lipid	1-(1-enyl-palmitoyl)-2-palmitoleoyl-GPC (P-16:0/16:1)*	-0.04	-0.25	0.17	0.68	0.86
Lipid	1-stearyl-GPC (O-18:0)*	0.06	-0.26	0.39	0.69	0.87
Lipid	1-linoleoyl-GPI (18:2)*	-0.13	-0.83	0.57	0.69	0.87
Lipid	12-HETE	0.21	-0.93	1.36	0.70	0.87
Lipid	2-hydroxypalmitate	-0.09	-0.56	0.38	0.70	0.87
Lipid	sphingomyelin (d18:2/18:1)*	-0.03	-0.20	0.13	0.70	0.87
Lipid	pregnenetriol sulfate*	-0.02	-0.11	0.08	0.70	0.87
Lipid	octadecadienedioate (C18:2-DC)*	-0.03	-0.21	0.14	0.70	0.87
Lipid	behenoyl dihydrosphingomyelin (d18:0/22:0)*	-0.04	-0.23	0.16	0.70	0.87
Lipid	2-palmitoyl-GPC (16:0)*	-0.07	-0.45	0.31	0.70	0.87
Lipid	1-oleoyl-GPC (18:1)	0.04	-0.18	0.26	0.70	0.87
Lipid	1-myristoylglycerol (14:0)	0.10	-0.45	0.65	0.71	0.87
Lipid	sphingomyelin (d18:0/20:0, d16:0/22:0)*	-0.04	-0.23	0.16	0.71	0.87
Lipid	N-stearoyltaurine	-0.07	-0.44	0.30	0.71	0.87
Lipid	3-hydroxyhexanoate	-0.04	-0.25	0.18	0.72	0.88
Lipid	1-palmitoleoyl-GPE (16:1)*	0.03	-0.14	0.19	0.72	0.88
Lipid	behenoyl sphingomyelin (d18:1/22:0)*	0.04	-0.17	0.24	0.72	0.88
Lipid	oleoyl-oleoyl-glycerol (18:1/18:1) [1]*	0.06	-0.31	0.43	0.72	0.88
Lipid	1-palmitoyl-2-stearoyl-GPC (16:0/18:0)	-0.08	-0.52	0.36	0.72	0.88
Lipid	glycerophosphoethanolamine	0.08	-0.38	0.53	0.72	0.88
Lipid	N-oleoylserine	-0.07	-0.47	0.34	0.72	0.88
Lipid	1-linolenoyl-GPC (18:3)*	-0.05	-0.33	0.23	0.72	0.88
Lipid	adipoylcarnitine (C6-DC)	-0.03	-0.18	0.13	0.72	0.88

Lipid	1-linolenoyl-GPE (18:3)*	0.04	-0.19	0.27	0.72	0.88
Lipid	1-linoleoylglycerol (18:2)	-0.17	-1.15	0.82	0.73	0.88
Lipid	acetoacetate	-0.08	-0.59	0.42	0.73	0.88
Lipid	2-aminooctanoate	-0.06	-0.40	0.29	0.73	0.88
Lipid	N-palmitoylserine	0.06	-0.28	0.39	0.73	0.88
Lipid	5alpha-androstan-3alpha,17beta-diol 17-glucuronide	-0.05	-0.37	0.27	0.74	0.88
Lipid	androstenediol (3beta,17beta) monosulfate (2)	-0.02	-0.14	0.10	0.74	0.89
Lipid	erucate (22:1n9)	-0.06	-0.44	0.32	0.75	0.89
Lipid	lignoceroyl sphingomyelin (d18:1/24:0)	-0.03	-0.23	0.17	0.75	0.89
Lipid	5alpha-androstan-3beta,17alpha-diol disulfate	-0.01	-0.05	0.04	0.75	0.89
Lipid	andro steroid monosulfate C19H28O6S (1)*	-0.01	-0.10	0.07	0.76	0.89
Lipid	phosphatidylcholine (18:0/20:2, 20:0/18:2)*	-0.05	-0.36	0.27	0.76	0.90
Lipid	1-palmitoyl-GPC (16:0)	0.04	-0.26	0.35	0.77	0.90
Lipid	sphingomyelin (d18:2/24:2)*	-0.04	-0.32	0.24	0.77	0.90
Lipid	17alpha-hydroxypregnenolone 3-sulfate	0.05	-0.29	0.39	0.77	0.90
Lipid	diacylglycerol (16:1/18:2 [2], 16:0/18:3 [1])*	0.03	-0.17	0.22	0.77	0.90
Lipid	1-palmitoyl-2-arachidonoyl-GPI (16:0/20:4)*	-0.08	-0.68	0.52	0.77	0.90
Lipid	1-margaroyl-2-oleoyl-GPC (17:0/18:1)*	-0.03	-0.29	0.22	0.78	0.91
Lipid	3-carboxy-4-methyl-5-propyl-2-furanpropanoate (CMPF)	-0.01	-0.11	0.08	0.78	0.91
Lipid	1-dihomo-linolenylglycerol (20:3)	0.16	-1.11	1.44	0.79	0.92
Lipid	pregnenediol disulfate (C21H34O8S2)*	-0.01	-0.08	0.06	0.79	0.92
Lipid	1-stearoyl-2-adrenoyl-GPC (18:0/22:4)*	-0.02	-0.20	0.16	0.79	0.92
Lipid	cortolone glucuronide (1)	-0.03	-0.30	0.23	0.79	0.92
Lipid	1-stearoyl-2-meadoyl-GPC (18:0/20:3n9)*	-0.02	-0.14	0.11	0.80	0.92
Lipid	1-stearoyl-2-oleoyl-GPI (18:0/18:1)*	-0.05	-0.51	0.41	0.80	0.92
Lipid	valerate (5:0)	-0.07	-0.63	0.50	0.80	0.92
Lipid	5alpha-pregnan-3beta,20alpha-diol disulfate	-0.01	-0.11	0.08	0.81	0.92
Lipid	1-behenoyl-GPC (22:0)	-0.03	-0.32	0.25	0.81	0.92
Lipid	malonate	-0.07	-0.66	0.53	0.81	0.92
Lipid	1-palmitoleoyl-2-linolenoyl-GPC (16:1/18:3)*	-0.04	-0.42	0.33	0.82	0.92

Lipid	1-stearoyl-2-arachidonoyl-GPC (18:0/20:4)	-0.02	-0.20	0.16	0.82	0.92
Lipid	oleoyl-arachidonoyl-glycerol (18:1/20:4) [2]*	-0.04	-0.45	0.36	0.82	0.92
Lipid	1-palmitoyl-GPE (16:0)	0.03	-0.22	0.27	0.82	0.92
Lipid	1-linoleoyl-GPC (18:2)	-0.05	-0.50	0.40	0.82	0.93
Lipid	1-oleoyl-GPI (18:1)	-0.08	-0.81	0.66	0.83	0.93
Lipid	palmitoylcholine	0.06	-0.57	0.70	0.83	0.93
Lipid	pregnenolone sulfate	-0.02	-0.25	0.20	0.83	0.93
Lipid	11beta-hydroxyandrosterone glucuronide	0.01	-0.10	0.12	0.84	0.93
Lipid	1-nonadecenoyl-GPC (19:1)*	0.03	-0.31	0.38	0.84	0.93
Lipid	margaroylcarnitine (C17)*	-0.03	-0.40	0.33	0.84	0.93
Lipid	linoleoyl-arachidonoyl-glycerol (18:2/20:4) [2]*	-0.02	-0.22	0.18	0.84	0.93
Lipid	tetrahydrocortisol sulfate (1)	-0.01	-0.13	0.11	0.85	0.94
Lipid	N-stearoylserine*	0.07	-0.69	0.83	0.85	0.94
Lipid	glycochenodeoxycholate glucuronide (1)	-0.01	-0.13	0.11	0.85	0.94
Lipid	4-methylhexanoylglutamine	-0.01	-0.18	0.15	0.85	0.94
Lipid	1-eicosapentaenoyl-GPC (20:5)*	0.02	-0.17	0.21	0.85	0.94
Lipid	hexanoylglutamine	-0.02	-0.25	0.21	0.86	0.94
Lipid	1-stearyl-2-arachidonoyl-GPC (O-18:0/20:4)*	0.02	-0.19	0.22	0.86	0.94
Lipid	5alpha-androstan-3alpha,17beta-diol disulfate	-0.01	-0.07	0.06	0.86	0.94
Lipid	1-palmitoyl-2-oleoyl-GPI (16:0/18:1)*	0.03	-0.36	0.43	0.86	0.94
Lipid	1-oleoyl-GPG (18:1)*	0.04	-0.42	0.50	0.86	0.94
Lipid	branched chain 14:0 dicarboxylic acid**	0.01	-0.12	0.14	0.86	0.94
Lipid	eicosenoylcarnitine (C20:1)*	-0.02	-0.27	0.23	0.87	0.94
Lipid	16alpha-hydroxy DHEA 3-sulfate	0.01	-0.08	0.10	0.87	0.94
Lipid	2S,3R-dihydroxybutyrate	0.02	-0.18	0.21	0.87	0.94
Lipid	3-hydroxyoleoylcarnitine	-0.04	-0.49	0.42	0.87	0.94
Lipid	hydroxypalmitoyl sphingomyelin (d18:1/16:0(OH))**	0.02	-0.23	0.27	0.87	0.94
Lipid	cis-3,4-methyleneheptanoylcarnitine	0.01	-0.15	0.18	0.88	0.95
Lipid	oleoyl-linoleoyl-glycerol (18:1/18:2) [2]	-0.01	-0.13	0.12	0.88	0.95
Lipid	1-palmitoyl-GPI (16:0)	0.04	-0.59	0.67	0.89	0.95

Lipid	nervonoylcarnitine (C24:1)*	-0.03	-0.45	0.40	0.89	0.96
Lipid	campesterol	-0.01	-0.24	0.21	0.90	0.96
Lipid	2-hydroxyglutarate	0.01	-0.22	0.25	0.90	0.96
Lipid	decadienedioic acid (C10:2-DC)**	0.01	-0.09	0.11	0.91	0.97
Lipid	N-stearoyl-sphingadienine (d18:2/18:0)*	0.01	-0.18	0.20	0.92	0.97
Lipid	beta-sitosterol	-0.01	-0.24	0.22	0.92	0.97
Lipid	1-docosahexaenoylglycerol (22:6)	-0.01	-0.32	0.29	0.92	0.97
Lipid	1-myristoyl-GPC (14:0)	0.01	-0.23	0.25	0.92	0.97
Lipid	4-cholesten-3-one	0.03	-0.65	0.71	0.93	0.97
Lipid	1-stearoyl-2-docosahexaenoyl-GPI (18:0/22:6)*	0.02	-0.46	0.50	0.93	0.97
Lipid	2-myristoyl-GPC (14:0)*	0.01	-0.25	0.27	0.93	0.97
Lipid	1-dihomo-linolenoyl-GPE (20:3n3 or 6)*	-0.01	-0.21	0.20	0.93	0.97
Lipid	5alpha-androstan-3beta,17beta-diol disulfate	0.00	-0.06	0.07	0.94	0.98
Lipid	2-hydroxyarachidate*	-0.01	-0.27	0.25	0.95	0.98
Lipid	1-docosahexaenoyl-GPC (22:6)*	0.01	-0.24	0.25	0.95	0.98
Lipid	2-hydroxysebacate	0.00	-0.20	0.19	0.96	0.99
Lipid	androstenediol (3alpha, 17alpha) monosulfate (2)	0.00	-0.08	0.08	0.97	0.99
Lipid	2-hydroxynervonate*	0.01	-0.61	0.62	0.98	0.99
Lipid	11beta-hydroxyetiocholanolone glucuronide*	0.00	-0.11	0.11	0.98	0.99
Lipid	nisinate (24:6n3)	0.01	-0.50	0.51	0.98	0.99
Lipid	1-eicosapentaenoyl-GPE (20:5)*	0.00	-0.17	0.17	0.99	1.00
Lipid	2-hydroxystearate	0.00	-0.55	0.56	0.99	1.00
NA	X-21353	-0.62	-0.85	-0.39	0.00	0.01
NA	X-23782	-0.46	-0.64	-0.27	0.00	0.01
NA	X-26106	-0.65	-1.02	-0.29	0.00	0.05
NA	X-12007	-0.11	-0.18	-0.05	0.00	0.05
NA	X-17351	-0.19	-0.31	-0.08	0.00	0.07
NA	X-12740	-0.12	-0.19	-0.05	0.00	0.07
NA	X-17690	-0.08	-0.13	-0.03	0.00	0.07
NA	X-16935	-0.09	-0.15	-0.03	0.00	0.09

NA	X-18913	-0.26	-0.43	-0.09	0.01	0.10
NA	X-11858	-0.05	-0.09	-0.02	0.01	0.12
NA	X-12283	-0.24	-0.42	-0.07	0.01	0.14
NA	X-21959	0.94	0.26	1.62	0.01	0.15
NA	X-21815	-0.07	-0.12	-0.02	0.01	0.15
NA	X-18921	-0.19	-0.34	-0.05	0.01	0.15
NA	X-23644	-0.09	-0.16	-0.02	0.01	0.16
NA	X-25371	-0.23	-0.42	-0.05	0.02	0.20
NA	X-17146	1.14	0.21	2.07	0.02	0.21
NA	X-11372	-0.22	-0.40	-0.04	0.02	0.21
NA	X-17438	-0.17	-0.32	-0.03	0.02	0.22
NA	X-24571	-0.09	-0.17	-0.02	0.02	0.22
NA	X-11850	-0.05	-0.09	-0.01	0.02	0.24
NA	X-12847	-0.07	-0.14	-0.01	0.03	0.24
NA	X-22834	-0.21	-0.38	-0.03	0.03	0.25
NA	X-13431	-0.41	-0.76	-0.05	0.03	0.26
NA	X-21821	-0.12	-0.22	-0.01	0.03	0.26
NA	X-12822	-0.18	-0.35	-0.02	0.03	0.30
NA	X-22143	-0.18	-0.36	-0.01	0.04	0.32
NA	X-26054	-0.26	-0.50	-0.01	0.04	0.33
NA	X-12013	-0.07	-0.14	0.00	0.04	0.33
NA	X-12818	-0.09	-0.17	0.00	0.05	0.34
NA	X-24337	-0.16	-0.33	0.00	0.05	0.35
NA	X-18888	-0.18	-0.36	0.00	0.05	0.35
NA	X-22509	-0.09	-0.18	0.00	0.05	0.35
NA	X-12851	-0.07	-0.14	0.00	0.05	0.35
NA	X-17354	-0.11	-0.21	0.00	0.05	0.35
NA	X-23680	-0.23	-0.47	0.00	0.05	0.35
NA	X-11861	-0.27	-0.55	0.01	0.05	0.36
NA	X-24522	-0.40	-0.82	0.01	0.05	0.36

NA	X-12410	-0.10	-0.21	0.00	0.06	0.36
NA	X-22776	-0.66	-1.35	0.03	0.06	0.37
NA	X-25520	-0.20	-0.41	0.02	0.07	0.39
NA	X-17365	-0.09	-0.20	0.01	0.07	0.39
NA	X-24352	-0.09	-0.19	0.01	0.07	0.40
NA	X-15728	-0.04	-0.08	0.00	0.07	0.40
NA	X-12306	-0.09	-0.19	0.01	0.07	0.40
NA	X-12411	-0.28	-0.59	0.03	0.08	0.40
NA	X-25419	-0.39	-0.82	0.05	0.08	0.40
NA	X-21285	-0.12	-0.24	0.01	0.08	0.40
NA	X-12713	-0.15	-0.32	0.02	0.08	0.40
NA	X-12462	0.62	-0.10	1.33	0.09	0.43
NA	X-23655	-0.46	-1.00	0.07	0.09	0.43
NA	X-17010	0.32	-0.05	0.69	0.09	0.43
NA	X-14056	-0.73	-1.59	0.13	0.09	0.44
NA	X-17325	-0.10	-0.23	0.02	0.10	0.46
NA	X-26109	-0.05	-0.10	0.01	0.10	0.46
NA	X-13553	-0.10	-0.22	0.02	0.10	0.47
NA	X-11299	-0.03	-0.06	0.01	0.10	0.47
NA	X-13728	-0.06	-0.14	0.02	0.11	0.48
NA	X-21788	-0.27	-0.60	0.07	0.11	0.48
NA	X-11787	-0.21	-0.47	0.05	0.11	0.49
NA	X-16576	0.22	-0.06	0.50	0.12	0.51
NA	X-21310	-0.24	-0.55	0.07	0.12	0.51
NA	X-21834	-0.05	-0.11	0.01	0.12	0.51
NA	X-13695	-0.19	-0.43	0.06	0.12	0.51
NA	X-21831	-0.22	-0.50	0.07	0.12	0.51
NA	X-21471	-0.14	-0.31	0.04	0.12	0.51
NA	X-24411	0.28	-0.09	0.65	0.13	0.51
NA	X-23157	-0.94	-2.19	0.31	0.13	0.51

NA	X-24306	-0.72	-1.70	0.26	0.14	0.52
NA	X-12714	-0.09	-0.22	0.03	0.14	0.52
NA	X-11378	-0.22	-0.52	0.08	0.14	0.53
NA	X-13844	-0.06	-0.14	0.02	0.14	0.53
NA	X-23665	-0.48	-1.15	0.19	0.15	0.54
NA	X-12126	-0.10	-0.24	0.04	0.15	0.54
NA	X-23276	0.33	-0.14	0.81	0.16	0.54
NA	X-17348	-0.02	-0.04	0.01	0.16	0.55
NA	X-25519	-0.45	-1.11	0.21	0.17	0.55
NA	X-17357	0.13	-0.06	0.32	0.17	0.56
NA	X-24418	0.09	-0.05	0.22	0.18	0.56
NA	X-26107	-0.22	-0.55	0.11	0.18	0.56
NA	X-12812	-0.08	-0.20	0.04	0.18	0.56
NA	X-11880	-0.17	-0.42	0.09	0.18	0.56
NA	X-15503	-0.13	-0.32	0.07	0.19	0.56
NA	X-12839	-0.20	-0.50	0.11	0.19	0.56
NA	X-24970	-0.22	-0.57	0.12	0.19	0.56
NA	X-21339	-0.08	-0.22	0.05	0.19	0.56
NA	X-17335	-0.22	-0.57	0.13	0.20	0.57
NA	X-13866	-0.08	-0.21	0.05	0.20	0.57
NA	X-21816	0.18	-0.11	0.46	0.20	0.57
NA	X-21442	0.03	-0.02	0.07	0.20	0.57
NA	X-12111	-0.13	-0.34	0.08	0.21	0.58
NA	X-25454	-0.46	-1.21	0.29	0.21	0.58
NA	X-11483	-0.03	-0.08	0.02	0.22	0.59
NA	X-24243	-0.17	-0.47	0.12	0.22	0.59
NA	X-13658	-0.03	-0.09	0.02	0.23	0.60
NA	X-21796	0.34	-0.24	0.92	0.23	0.60
NA	X-26111	-0.02	-0.06	0.02	0.23	0.60
NA	X-25279	-0.06	-0.17	0.04	0.24	0.60

NA	X-21410	-0.14	-0.39	0.11	0.24	0.60
NA	X-11849	-0.01	-0.04	0.01	0.24	0.61
NA	X-23583	-0.21	-0.58	0.16	0.25	0.61
NA	X-11478	-0.08	-0.21	0.06	0.25	0.61
NA	X-12798	-0.07	-0.18	0.05	0.25	0.61
NA	X-26062	-0.16	-0.44	0.12	0.25	0.61
NA	X-23739	-0.29	-0.82	0.23	0.25	0.62
NA	X-16938	0.22	-0.18	0.62	0.26	0.62
NA	X-12707	-0.25	-0.70	0.21	0.26	0.63
NA	X-21319	0.18	-0.15	0.51	0.26	0.63
NA	X-11407	0.16	-0.14	0.46	0.26	0.63
NA	X-23587	-0.16	-0.45	0.13	0.27	0.63
NA	X-12731	-0.08	-0.22	0.07	0.28	0.64
NA	X-21258	-0.03	-0.10	0.03	0.28	0.64
NA	X-23659	0.19	-0.18	0.56	0.28	0.64
NA	X-12104	-0.18	-0.54	0.17	0.29	0.64
NA	X-12112	0.14	-0.13	0.42	0.29	0.64
NA	X-12830	-0.05	-0.14	0.05	0.29	0.64
NA	X-25422	-0.07	-0.20	0.07	0.30	0.65
NA	X-12816	-0.01	-0.03	0.01	0.30	0.65
NA	X-23780	-0.17	-0.52	0.17	0.30	0.65
NA	X-24953	-0.14	-0.44	0.15	0.31	0.66
NA	X-17355	-0.18	-0.55	0.19	0.32	0.66
NA	X-12729	-0.05	-0.15	0.05	0.32	0.67
NA	X-21842	-0.06	-0.18	0.06	0.32	0.67
NA	X-11308	-0.07	-0.20	0.07	0.33	0.67
NA	X-21742	-0.01	-0.04	0.01	0.33	0.67
NA	X-24295	-0.33	-1.02	0.37	0.33	0.67
NA	X-26119	-0.05	-0.16	0.06	0.33	0.67
NA	X-12726	-0.17	-0.56	0.21	0.35	0.69

NA	X-16397	0.16	-0.19	0.50	0.35	0.69
NA	X-11612	-0.09	-0.29	0.11	0.35	0.69
NA	X-11843	-0.03	-0.10	0.04	0.36	0.69
NA	X-24736	0.15	-0.19	0.50	0.36	0.69
NA	X-11315	0.06	-0.08	0.20	0.37	0.70
NA	X-24334	-0.07	-0.22	0.09	0.37	0.70
NA	X-17367	-0.13	-0.43	0.17	0.38	0.70
NA	X-18345	-0.07	-0.22	0.09	0.38	0.70
NA	X-23593	0.13	-0.18	0.44	0.38	0.70
NA	X-24494	0.06	-0.08	0.20	0.38	0.70
NA	X-24328	-0.05	-0.18	0.07	0.38	0.70
NA	X-17676	-0.06	-0.21	0.09	0.38	0.70
NA	X-13726	-0.02	-0.08	0.03	0.38	0.70
NA	X-17685	-0.02	-0.06	0.03	0.38	0.70
NA	X-18901	-0.03	-0.12	0.05	0.38	0.70
NA	X-16649	0.15	-0.22	0.52	0.39	0.70
NA	X-23974	-0.17	-0.57	0.24	0.39	0.70
NA	X-21383	0.18	-0.27	0.64	0.41	0.71
NA	X-17328	-0.08	-0.30	0.13	0.41	0.71
NA	X-21312	0.06	-0.09	0.21	0.42	0.71
NA	X-11847	0.03	-0.04	0.09	0.43	0.73
NA	X-25810	-0.29	-1.07	0.48	0.43	0.73
NA	X-19183	0.14	-0.23	0.50	0.44	0.73
NA	X-23787	-0.04	-0.14	0.06	0.44	0.73
NA	X-24951	-0.16	-0.61	0.29	0.45	0.73
NA	X-21470	-0.03	-0.10	0.05	0.45	0.73
NA	X-24757	0.09	-0.16	0.33	0.45	0.73
NA	X-25417	-0.10	-0.39	0.18	0.46	0.74
NA	X-21840	0.02	-0.03	0.06	0.46	0.74
NA	X-24947	-0.02	-0.09	0.05	0.47	0.74

NA	X-23639	0.07	-0.13	0.28	0.47	0.74
NA	X-12906	0.11	-0.20	0.42	0.47	0.74
NA	X-24949	-0.04	-0.17	0.08	0.47	0.74
NA	X-12844	-0.06	-0.22	0.11	0.48	0.74
NA	X-18899	0.19	-0.38	0.77	0.48	0.75
NA	X-12101	0.04	-0.07	0.15	0.49	0.75
NA	X-18779	-0.24	-0.97	0.49	0.49	0.75
NA	X-21607	-0.08	-0.34	0.17	0.49	0.76
NA	X-24544	-0.03	-0.11	0.06	0.50	0.76
NA	X-11632	-0.10	-0.41	0.21	0.50	0.76
NA	X-24812	-0.07	-0.27	0.14	0.51	0.77
NA	X-23666	0.17	-0.38	0.72	0.51	0.77
NA	X-25420	-0.10	-0.41	0.22	0.51	0.77
NA	X-21441	-0.03	-0.12	0.06	0.53	0.77
NA	X-21752	-0.01	-0.05	0.02	0.53	0.77
NA	X-11795	-0.07	-0.30	0.16	0.53	0.77
NA	X-21851	-0.05	-0.22	0.12	0.53	0.77
NA	X-24972	0.09	-0.21	0.39	0.54	0.78
NA	X-12015	0.05	-0.13	0.24	0.54	0.78
NA	X-14082	-0.09	-0.40	0.22	0.54	0.78
NA	X-15245	0.12	-0.29	0.53	0.55	0.78
NA	X-18886	0.04	-0.10	0.18	0.55	0.79
NA	X-21286	-0.07	-0.31	0.17	0.55	0.79
NA	X-14904	-0.15	-0.69	0.38	0.55	0.79
NA	X-24637	-0.01	-0.05	0.03	0.55	0.79
NA	X-17612	0.18	-0.46	0.82	0.55	0.79
NA	X-24728	0.10	-0.27	0.48	0.56	0.80
NA	X-25343	-0.15	-0.67	0.38	0.57	0.80
NA	X-15674	0.20	-0.55	0.95	0.57	0.80
NA	X-24556	-0.09	-0.44	0.25	0.58	0.81

NA	X-14939	-0.04	-0.18	0.10	0.59	0.81
NA	X-15220	-0.18	-0.87	0.51	0.59	0.81
NA	X-24475	-0.08	-0.40	0.24	0.60	0.81
NA	X-17682	0.08	-0.24	0.40	0.60	0.81
NA	X-25937	-0.03	-0.13	0.08	0.60	0.81
NA	X-11381	-0.03	-0.17	0.11	0.61	0.82
NA	X-18922	-0.03	-0.15	0.09	0.62	0.82
NA	X-17346	-0.07	-0.35	0.21	0.62	0.82
NA	X-17654	-0.03	-0.18	0.11	0.62	0.82
NA	X-26097	-0.13	-0.68	0.42	0.62	0.82
NA	X-25957	-0.21	-1.10	0.68	0.62	0.82
NA	X-13507	-0.05	-0.28	0.18	0.63	0.83
NA	X-23654	-0.07	-0.39	0.25	0.63	0.83
NA	X-12117	0.09	-0.30	0.47	0.64	0.83
NA	X-19141	-0.03	-0.14	0.09	0.64	0.83
NA	X-16087	0.04	-0.16	0.25	0.65	0.84
NA	X-16580	-0.05	-0.30	0.19	0.66	0.85
NA	X-21845	-0.05	-0.28	0.19	0.67	0.85
NA	X-21364	-0.02	-0.11	0.07	0.68	0.86
NA	X-12456	-0.02	-0.11	0.08	0.69	0.86
NA	X-13007	0.08	-0.33	0.48	0.69	0.87
NA	X-07765	-0.01	-0.07	0.05	0.70	0.87
NA	X-12100	-0.06	-0.39	0.27	0.70	0.87
NA	X-19299	0.07	-0.30	0.43	0.70	0.87
NA	X-16964	0.13	-0.57	0.82	0.71	0.87
NA	X-11979	0.07	-0.30	0.43	0.71	0.87
NA	X-24588	-0.04	-0.28	0.20	0.72	0.88
NA	X-21327	0.11	-0.53	0.74	0.72	0.88
NA	X-24422	0.06	-0.30	0.43	0.72	0.88
NA	X-23997	-0.05	-0.36	0.26	0.74	0.88

NA	X-13688	-0.03	-0.23	0.17	0.74	0.89
NA	X-25267	0.08	-0.44	0.61	0.75	0.89
NA	X-11470	-0.02	-0.15	0.11	0.75	0.89
NA	X-11444	0.02	-0.09	0.12	0.75	0.89
NA	X-12708	-0.06	-0.51	0.39	0.78	0.91
NA	X-16124	0.01	-0.05	0.06	0.79	0.92
NA	X-24549	-0.08	-0.76	0.59	0.80	0.92
NA	X-24462	-0.06	-0.53	0.42	0.80	0.92
NA	X-25265	0.13	-0.94	1.19	0.80	0.92
NA	X-24307	-0.05	-0.52	0.41	0.80	0.92
NA	X-25271	-0.03	-0.31	0.25	0.81	0.92
NA	X-12544	0.01	-0.07	0.09	0.82	0.92
NA	X-12680	-0.02	-0.16	0.13	0.82	0.92
NA	X-22508	-0.05	-0.50	0.41	0.83	0.93
NA	X-25948	-0.05	-0.55	0.45	0.84	0.93
NA	X-22520	-0.02	-0.20	0.16	0.84	0.93
NA	X-12026	-0.03	-0.36	0.30	0.85	0.94
NA	X-21467	-0.01	-0.14	0.12	0.85	0.94
NA	X-15461	-0.12	-1.47	1.24	0.86	0.94
NA	X-12718	-0.03	-0.42	0.35	0.86	0.94
NA	X-23662	0.01	-0.06	0.07	0.86	0.94
NA	X-12216	-0.01	-0.14	0.12	0.88	0.95
NA	X-19438	-0.06	-0.85	0.73	0.88	0.95
NA	X-13729	-0.01	-0.13	0.11	0.88	0.95
NA	X-12261	0.00	-0.03	0.03	0.88	0.95
NA	X-22162	-0.02	-0.42	0.37	0.89	0.96
NA	X-24669	0.01	-0.23	0.26	0.90	0.96
NA	X-10458	-0.02	-0.35	0.32	0.91	0.97
NA	X-15486	0.01	-0.16	0.17	0.93	0.97
NA	X-13684	-0.01	-0.25	0.23	0.93	0.97

NA	X-12221	-0.01	-0.26	0.24	0.93	0.97
NA	X-18887	-0.01	-0.19	0.18	0.94	0.98
NA	X-17653	-0.01	-0.20	0.18	0.94	0.98
NA	X-21733	-0.01	-0.19	0.18	0.95	0.98
NA	X-12193	0.00	-0.18	0.19	0.96	0.99
NA	X-26108	0.02	-0.70	0.74	0.96	0.99
NA	X-24546	0.00	-0.10	0.09	0.96	0.99
NA	X-11852	0.00	-0.06	0.06	0.97	0.99
NA	X-25433	0.00	-0.16	0.17	0.97	0.99
NA	X-25790	0.00	-0.29	0.30	0.98	0.99
NA	X-18935	0.00	-0.07	0.08	0.98	0.99
NA	X-22771	0.00	-0.33	0.33	0.98	0.99
NA	X-12407	0.00	-0.30	0.30	0.99	0.99
NA	X-23636	0.00	-0.54	0.54	0.99	1.00
NA	X-21736	0.00	-0.15	0.15	1.00	1.00
NA	X-25457	0.00	-0.11	0.11	1.00	1.00
NA	X-21661	0.00	-0.04	0.04	1.00	1.00
Nucleotide	2'-O-methyluridine	-0.16	-0.35	0.02	0.08	0.41
Nucleotide	xanthosine	-0.27	-0.61	0.07	0.11	0.49
Nucleotide	N6-carbamoylthreonyladenosine	-0.15	-0.35	0.05	0.12	0.51
Nucleotide	2'-deoxyuridine	0.29	-0.12	0.70	0.15	0.54
Nucleotide	adenosine	-0.40	-0.96	0.17	0.15	0.54
Nucleotide	pseudouridine	-0.26	-0.62	0.11	0.16	0.54
Nucleotide	guanosine	-0.45	-1.11	0.20	0.16	0.54
Nucleotide	5,6-dihydrouridine	-0.17	-0.43	0.08	0.17	0.56
Nucleotide	uridine	-0.18	-0.48	0.12	0.22	0.59
Nucleotide	allantoin	-0.30	-0.80	0.20	0.22	0.59
Nucleotide	N2,N2-dimethylguanosine	-0.33	-0.95	0.29	0.28	0.64
Nucleotide	adenosine 3',5'-cyclic monophosphate (cAMP)	0.36	-0.33	1.04	0.28	0.64
Nucleotide	inosine	-0.31	-0.92	0.30	0.30	0.65

Nucleotide	N6-methyladenosine	-0.18	-0.56	0.20	0.32	0.67
Nucleotide	N6-succinyladenosine	0.15	-0.20	0.49	0.38	0.70
Nucleotide	orotidine	-0.08	-0.26	0.11	0.39	0.70
Nucleotide	xanthine	-0.06	-0.22	0.09	0.39	0.70
Nucleotide	cytidine	-0.26	-0.90	0.37	0.40	0.70
Nucleotide	N-acetyl-beta-alanine	0.15	-0.22	0.51	0.41	0.71
Nucleotide	5,6-dihydrouracil	0.19	-0.32	0.71	0.43	0.73
Nucleotide	adenine	0.18	-0.32	0.67	0.45	0.73
Nucleotide	adenosine 5'-monophosphate (AMP)	0.22	-0.45	0.89	0.49	0.75
Nucleotide	3-ureidopropionate	0.09	-0.18	0.37	0.49	0.75
Nucleotide	3-methylcytidine	-0.12	-0.52	0.28	0.53	0.78
Nucleotide	thymidine	-0.09	-0.39	0.21	0.54	0.78
Nucleotide	N1-methylinosine	-0.10	-0.43	0.23	0.54	0.78
Nucleotide	N4-acetylcytidine	0.12	-0.41	0.65	0.64	0.83
Nucleotide	5,6-dihydrothymine	-0.17	-0.95	0.60	0.64	0.83
Nucleotide	orotate	-0.06	-0.33	0.22	0.65	0.84
Nucleotide	7-methylguanine	-0.14	-0.79	0.52	0.66	0.85
Nucleotide	dihydroorotate	-0.05	-0.36	0.26	0.73	0.88
Nucleotide	5-methyluridine (ribothymidine)	-0.02	-0.13	0.10	0.75	0.89
Nucleotide	N1-methyladenosine	-0.06	-0.44	0.33	0.76	0.90
Nucleotide	uracil	0.05	-0.37	0.48	0.80	0.92
Nucleotide	3-aminoisobutyrate	0.02	-0.17	0.21	0.84	0.93
Nucleotide	urate	-0.02	-0.32	0.28	0.87	0.94
Nucleotide	2'-O-methylcytidine	-0.03	-0.75	0.69	0.93	0.97
Nucleotide	3-(3-amino-3-carboxypropyl)uridine*	-0.01	-0.28	0.27	0.96	0.99
Nucleotide	hypoxanthine	0.00	-0.84	0.85	0.99	1.00
Partially Characterized Molecules	branched-chain, straight-chain, or cyclopropyl 10:1 fatty acid (1)*	-0.22	-0.37	-0.06	0.01	0.15

Partially Characterized Molecules	pentose acid*	-0.16	-0.33	0.00	0.05	0.35
Partially Characterized Molecules	glycine conjugate of C10H12O2*	-0.24	-0.56	0.08	0.13	0.51
Partially Characterized Molecules	glycine conjugate of C10H14O2 (1)*	-0.11	-0.25	0.04	0.13	0.52
Partially Characterized Molecules	bilirubin degradation product, C16H18N2O5 (4)**	-0.26	-0.65	0.14	0.19	0.56
Partially Characterized Molecules	branched-chain, straight-chain, or cyclopropyl 12:1 fatty acid*	-0.45	-1.15	0.26	0.19	0.56
Partially Characterized Molecules	glutamine_degradant*	-0.07	-0.17	0.04	0.21	0.58
Partially Characterized Molecules	bilirubin degradation product, C16H18N2O5 (2)**	-0.10	-0.28	0.08	0.24	0.60
Partially Characterized Molecules	glucuronide of C10H18O2 (1)*	-0.08	-0.27	0.11	0.38	0.70
Partially Characterized Molecules	glucuronide of C10H18O2 (7)*	0.05	-0.07	0.17	0.42	0.71
Partially Characterized Molecules	bilirubin degradation product, C17H18N2O4 (3)**	-0.12	-0.42	0.18	0.42	0.71
Partially Characterized Molecules	bilirubin degradation product, C17H20N2O5 (1)**	-0.21	-0.77	0.36	0.45	0.73
Partially Characterized Molecules	metabolonic lactone sulfate	-0.02	-0.08	0.04	0.45	0.73
Partially Characterized Molecules	glutamine conjugate of C6H10O2 (2)*	0.05	-0.09	0.18	0.45	0.73
Partially Characterized Molecules	glutamine conjugate of C6H10O2 (1)*	0.05	-0.09	0.20	0.46	0.74

Partially Characterized Molecules	bilirubin degradation product, C17H20N2O5 (2)**	-0.16	-0.67	0.35	0.52	0.77
Partially Characterized Molecules	bilirubin degradation product, C16H18N2O5 (1)**	0.07	-0.15	0.29	0.52	0.77
Partially Characterized Molecules	GlcNAc sulfate conjugate of C21H34O2 steroid**	-0.03	-0.14	0.08	0.54	0.78
Partially Characterized Molecules	bilirubin degradation product, C16H18N2O5 (3)**	-0.06	-0.29	0.18	0.62	0.82
Partially Characterized Molecules	glutamine conjugate of C7H12O2*	0.03	-0.12	0.19	0.67	0.85
Partially Characterized Molecules	bilirubin degradation product, C17H18N2O4 (2)**	-0.06	-0.36	0.24	0.68	0.86
Partially Characterized Molecules	bilirubin degradation product, C17H18N2O4 (1)**	0.02	-0.30	0.34	0.89	0.96
Peptide	gamma-glutamylmethionine	-0.48	-0.94	-0.02	0.04	0.32
Peptide	4-hydroxyphenylacetylglutamine	-0.11	-0.22	0.00	0.04	0.33
Peptide	isoleucylglycine	-0.39	-0.80	0.01	0.06	0.36
Peptide	gamma-glutamyl-alpha-lysine	-0.44	-0.94	0.05	0.08	0.40
Peptide	gamma-glutamyltryptophan	-0.29	-0.66	0.09	0.12	0.51
Peptide	phenylacetylglutamate	-0.12	-0.28	0.04	0.14	0.52
Peptide	3-hydroxyphenylacetoylglutamine	-0.07	-0.17	0.03	0.15	0.53
Peptide	gamma-glutamyltyrosine	-0.19	-0.46	0.08	0.15	0.54
Peptide	cyclo(leu-pro)	-0.09	-0.23	0.04	0.16	0.55
Peptide	gamma-glutamylhistidine	-0.22	-0.55	0.12	0.18	0.56
Peptide	gamma-glutamylleucine	-0.11	-0.31	0.08	0.23	0.60
Peptide	gamma-glutamylisoleucine*	-0.13	-0.36	0.09	0.23	0.60
Peptide	gamma-glutamylthreonine	-0.14	-0.39	0.10	0.23	0.60
Peptide	fibrinopeptide B (1-13)**	1.03	-0.74	2.80	0.23	0.60
Peptide	fibrinopeptide A, des-ala(1)*	0.52	-0.41	1.45	0.25	0.61

Peptide	gamma-glutamylvaline	-0.08	-0.23	0.07	0.28	0.64
Peptide	isoleucylhydroxyproline*	-0.14	-0.45	0.17	0.34	0.68
Peptide	leucylglycine	-0.28	-0.93	0.37	0.38	0.70
Peptide	leucylhydroxyproline*	-0.08	-0.30	0.15	0.47	0.74
Peptide	gamma-glutamylglycine	-0.06	-0.26	0.15	0.57	0.80
Peptide	pyroglutamylvaline	0.07	-0.20	0.34	0.58	0.81
Peptide	gamma-glutamylglutamate	0.06	-0.16	0.27	0.58	0.81
Peptide	gamma-glutamylglutamine	-0.08	-0.52	0.35	0.69	0.86
Peptide	N,N-dimethyl-pro-pro	0.07	-0.45	0.59	0.77	0.90
Peptide	gamma-glutamylcitrulline*	-0.07	-0.81	0.66	0.83	0.93
Peptide	cyclo(pro-val)	0.02	-0.16	0.19	0.84	0.93
Peptide	phenylacetylcarnitine	0.00	-0.07	0.06	0.92	0.97
Peptide	phenylacetylglutamine	0.01	-0.16	0.17	0.94	0.98
Peptide	gamma-glutamylphenylalanine	-0.01	-0.41	0.39	0.96	0.99
Peptide	bradykinin, des-arg(9)	0.02	-0.76	0.79	0.97	0.99

Table S4

Mean change in metabolite level in all patients following exenatide infusion (PAH and CTEPH)
